# Discovery and Evaluation of Thiazinoquinones as Anti-Protozoal Agents

**DOI:** 10.3390/md11093472

**Published:** 2013-09-09

**Authors:** Cary F. C. Lam, A. Norrie Pearce, Shen H. Tan, Marcel Kaiser, Brent R. Copp

**Affiliations:** 1 School of Chemical Sciences, University of Auckland, Private Bag 92019, Auckland 1142, New Zealand; E-Mails: cf.lam@auckland.ac.nz (C.F.C.L.); n.pearce@auckland.ac.nz (A.N.P.); shen.tan@rsc.anu.edu.au (S.H.T.); 2 Swiss Tropical and Public Health Institute, Socinstrasse 57, PO Box, Basel CH-4002, Switzerland; E-Mail: marcel.kaiser@unibas.ch; 3 University of Basel, Basel CH-4003, Switzerland

**Keywords:** marine natural products, protozoa, malaria, *Plasmodium falciparum*, *Trypanosoma brucei rhodesiense*, quinone, dioxothiazine, alkaloid

## Abstract

Pure compound screening has identified the dioxothiazino-quinoline-quinone ascidian metabolite ascidiathiazone A (**2**) to be a moderate growth inhibitor of *Trypanosoma brucei rhodesiense* (IC_50_ 3.1 μM) and *Plasmodium falciparum* (K1 dual drug resistant strain) (IC_50_ 3.3 μM) while exhibiting low levels of cytotoxicity (L6, IC_50_ 167 μM). A series of C-7 amide and Δ^2(3)^ analogues were prepared that explored the influence of lipophilicity and oxidation state on observed anti-protozoal activity and selectivity. Little variation in anti-malarial potency was observed (IC_50_ 0.62–6.5 μM), and no correlation was apparent between anti-malarial and anti-*T. brucei* activity. Phenethylamide **7e** and Δ^2(3)^-glycine analogue **8k** exhibited similar anti-*Pf* activity to **2** but with slightly enhanced selectivity (SI 72 and 93, respectively), while Δ^2(3)^-phenethylamide **8e** (IC_50_ 0.67 μM, SI 78) exhibited improved potency and selectivity towards *T. brucei rhodesiense* compared to the natural product hit. A second series of analogues were prepared that replaced the quinoline ring of **2** with benzofuran or benzothiophene moieties. While esters **10a**/**10b** and **15** were once again found to exhibit cytotoxicity, carboxylic acid analogues exhibited potent anti-*Pf* activity (IC_50_ 0.34–0.035 μM) combined with excellent selectivity (SI 560–4000). *In vivo* evaluation of a furan carboxylic acid analogue against *P. berghei* was undertaken, demonstrating 85.7% and 47% reductions in parasitaemia with ip or oral dosing respectively.

## 1. Introduction

Natural products have historically played an important role in the discovery of new treatments for malaria [1]. From quinine and artemisinin [2,3] starting points, a diverse range of anti-malarials have been developed and have been the mainstay of frontline treatment for decades. Unfortunately with time has come loss of therapeutic efficacy due to the growing prevalence of drug resistant strains [4]. In the hunt for novel scaffolds from which to develop the next generation of anti-malarials, a structurally-diverse array of natural products, including those obtained from marine organisms, have been reported to exhibit activity towards *Plasmodium falciparum* [5,6,7].

**Figure 1:**
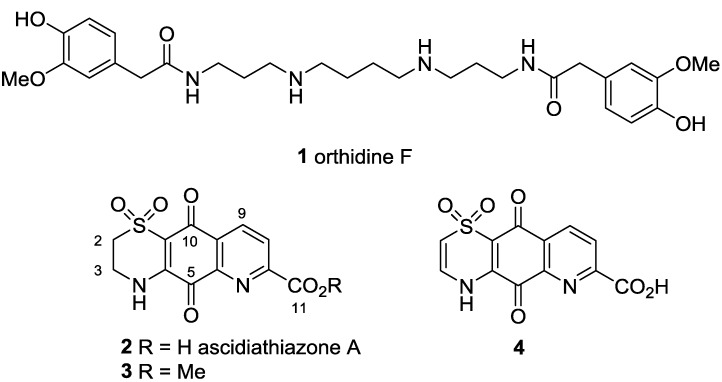
Structures of orthidine F (**1**), ascidiathiazone A (**2**) and analogues **3** and **4**.

As part of our own continuing search for new leads for the development of treatments for neglected human diseases [8,9,10,11,12] we have screened a library of synthesized and isolated marine natural products against a panel of four parasitic protozoa: *Trypanosoma brucei rhodesiense*, *Trypanosoma cruzi*, *Leishmania donovani* and *Plasmodium falciparum* K1 dual drug-resistant strain, with concurrent counter-screening for toxicity towards the non-malignant L6 rat myoblast cell line. We recently disclosed details of the first hit from this screen, the previously reported anti-inflammatory polyamine diamide ascidian metabolite orthidine F (**1**) [13,14,15] (Figure 1).

A second series of hits identified in this screening program were ascidiathiazone A (**2**), also previously reported by us as an anti-inflammatory alkaloid from a New Zealand ascidian, and synthetic analogues **3** and **4** [16]. The anti-protozoal evaluation of **2** (Table 1, entry 1) established the natural product to be a moderately potent *in vitro* growth inhibitor of *P. falciparum* K1 strain (IC_50_ 3.3 μM) and *Trypanosoma brucei rhodesiense* (IC_50_ 3.1 μM) while being effectively inactive towards *T. cruzi* and *Leishmania donovani* and exhibiting low levels of cytotoxicity against a mammalian cell-line (L6, IC_50_ 170 μM). Similar levels of potency and selectivity were observed for ester **3** (Table 1, entry 2), while Δ^2(3)^ analogue **4** (Table 1, entry 3) exhibited more potent anti-malarial activity (IC_50_ 0.6 μM) with enhanced selectivity (SI *Pf* 300). Herein we report the results of a preliminary structure-activity relationship study investigating the influence of C-2 amide functionalization and thiazine-Δ^2(3)^ oxidation on the biological activity of **2**. In addition, we report that novel furan and thiophene analogues of **2** exhibit potent *in vitro* anti-malarial activity and that one analogue exhibits *in vivo* activity towards *P. berghei*.

**Table 1:** Anti-protozoal, cytotoxic activities and clogP values of **2**–**4**, **7a**–**h**, **j**, **8a**–**k**.

Entry	Compound	IC_50_ (μM) ^a^					SI *Pf* ^g^	clogP ^h^
		*T. b. rhod.* ^b^	*T. cruzi* ^c^	*L. don.* ^d^	*P. falc.* K1 ^e^	L6 ^f^		
1	**2**	3.1	>290	270	3.3	170	50	−1.1 ± 1.1
2	**3**	6.6	180	31	2.5	140	56	−0.5 ± 0.9
3	**4**	4.0	>290	190	0.60	180	300	−1.1 ± 1.0
4	**7a**	5.5	63	>280	0.94	24	26	0.3 ± 0.5
5	**7b**	1.8	15	29	0.62	23	37	0.8 ± 0.6
6	**7c**	3.9	15	48	1.1	12	10	2.3 ± 0.6
7	**7d**	1.9	43	21	1.1	15	14	0.6 ± 0.5
8	**7e**	2.4	140	160	1.5	110	72	0.9 ± 0.5
9	**7f**	2.4	27	47	1.4	13	10	1.4 ± 0.4
10	**7g**	3.4	41	83	1.6	24	15	2.1 ± 0.6
11	**7h**	>150	53	170	2.4	41	17	−0.6 ± 0.7
12	**7j**	120	250	>260	3.4	110	31	−0.9 ± 0.5
13	**8a**	3.7	63	>280	0.70	23	33	0.3 ± 0.8
14	**8b**	3.6	48	>270	1.5	4.8	3	0.8 ± 0.8
15	**8c**	2.4	42	53	0.98	6.5	6	2.2 ± 0.8
16	**8d**	4.2	160	>250	4.7	34	7	0.8 ± 0.6
17	**8e**	0.67	140	160	6.5	52	8	0.9 ± 0.7
18	**8f**	5.9	59	>240	1.2	6.5	5	1.3 ± 0.7
19	**8g**	2.5	42	150	1.1	4.9	4	2.1 ± 0.7
20	**8h**	10	150	230	1.7	50	29	−0.6 ± 0.8
21	**8i**	13	140	150	1.5	99	67	−1.0 ± 0.9
22	**8j**	35	160	220	1.8	100	57	−1.1 ± 1.0
23	**8k**	42	160	>280	1.2	110	93	−1.2 ± 0.7
	Melarsoprol ^i^	0.005						
	Benznidazole ^i^		1.8					
	Miltefosine ^i^			0.53				
	Chloroquine ^i^				0.28			
	Podophyllotoxin ^i^					0.019		

^a^ IC_50_ values reported are the average of two independent assays. Assay protocols are described in [5]; ^b^ *Trypanosoma brucei rhodesiense*, STIB 900 strain, trypomastigotes stage; ^c^ *Trypanosoma cruzi*, Tulahuen C4 strain, amastigotes stage; ^d^ *Leishmania donovani*, MHOM-ET-67/L82 strain, amastigote/axenic stage; ^e^ *Plasmodium falciparum*, K1 strain, IEF stage; ^f^ L6 rat skeletal myoblast cell line; ^g^ Selectivity index for *P. falciparum* = IC_50_ L6/IC_50_ *Pf*; ^h^ cLogP calculated using ALOGPS 2.1, as described in [17,18]; ^i^ Melarsoprol, benznidazole, miltefosine, chloroquine and podophyllotoxin were used as positive controls.

## 2. Results and Discussion

### 2.1. Chemistry

**Scheme 1:**
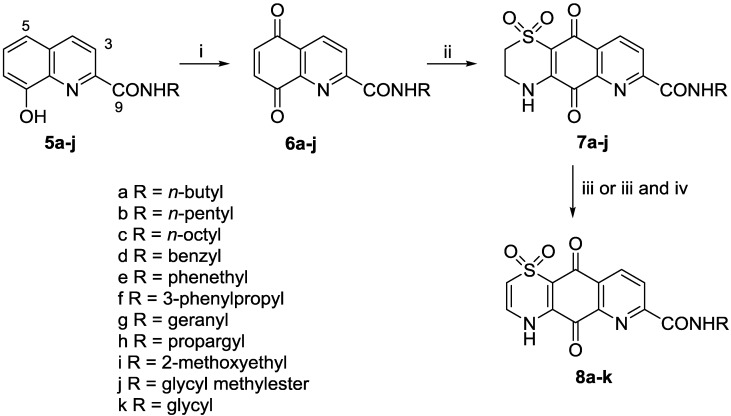
General reaction sequence for the preparation of analogues **7a**–**j** and **8a**–**k**. *Reagents and conditions*: (i) PIFA (2–3 equiv.), MeCN/H_2_O, 0 °C, 20 min; (ii) Hypotaurine (0.8 equiv.), CeCl_3_·7H_2_O, MeCN/EtOH, rt, 2 days; (iii) 2 N NaOH, DMF, rt, 2 h; (iv) SOCl_2_, MeOH, 0 °C then rt, then 65 °C, 2 h, 93% yield.

We undertook a preliminary structure-activity relationship study to explore the effect of carboxylic acid functionalization and thiazine ring oxidation state towards the observed anti-protozoal activity of **2**. Efforts to directly prepare amide derivatives of **2** by reaction of the synthesized natural product [16] with various amines in the presence of peptide coupling reagents, led to the formation of complex product mixtures and low yields (data not shown). Instead we made use of a longer four step reaction sequence (Scheme 1). Commercially available 8-hydroxyquinoline-2-carboxylic acid was converted to amides **5a**–**5j** by reaction with the appropriate amine using PyBOP as the coupling agent in DMF. Subsequent oxidation using PIFA (phenyliodine bis(trifluoroacetate)) in MeCN/H_2_O yielded unstable quinones **6a**–**6j**.

Previous studies by ourselves [16,19] and others [20,21] have found that hypotaurine addition to quinones typically yields a mixture of regio-isomeric thiazine adducts. In the present study, we found that slow addition of a dilute solution of hypotaurine in MeCN/EtOH at room temperature afforded, after filtration and washing, analogues **7a**–**7j** in yields of 14%, 27%, 57%, 17%, 49%, 57%, 29%, 29%, 26% and 20%, respectively. The regio-isomeric identity of the product in each case was established by 2D-NMR data, particularly HMBC experiments optimized for 8.3 and 2.0 Hz, which showed correlations from H-9 to quinone C-10 (8.3 Hz) and from NH-4 to quinone C-5 (2.0 Hz) [16]. Reaction of each of **7a**–**7j** with 2 N NaOH in DMF for 2 h [16] afforded the desired hydrolysed and autoxidised Δ^2(3)^-thiazine analogues **8a**–**8i** and **8k** in variable yield (30%–83%). In the specific case of the glycine methylester **7j** the product of this reaction was the Δ^2(3)^-thiazino carboxylic acid **8k**, methylation of which (SOCl_2_, MeOH, 93% yield) afforded ester **8j**.

**Scheme 2:**
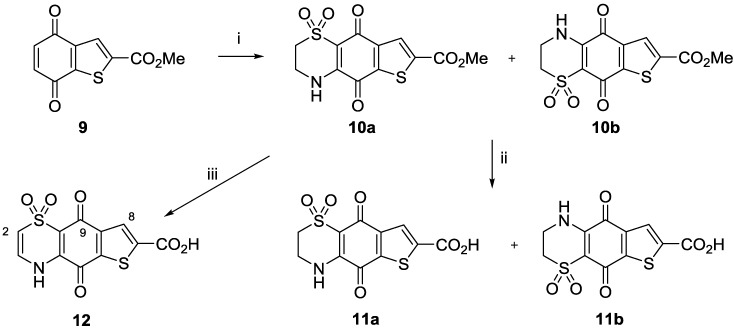
Preparation of thiophene analogues **10a**/**10b**, **11a**/**11b** and **12**. *Reagents and conditions*: (i) Hypotaurine (1 equiv.), CeCl_3_·7H_2_O (1 equiv.), MeCN/EtOH, rt, 2 days, 18% yield (**10a** + **10b**); (ii) conc. HCl, rt, 5 h, 57% yield (**11a** + **11b**); (iii) 1 N NaOH, EtOAc, rt, 1 h, 78% yield.

Thiophene analogues of ascidiathiazone A were prepared (Scheme 2) starting from the literature quinone **9** [22]. Reaction with hypotaurine yielded two isomeric products **10a** and **10b** in a ratio of 1:0.3, as determined by NMR. Despite extensive attempts using chromatography, the isomers could not be separated and so were used as a mixture in the following steps. The regio-isomeric identity of **10a** and **10b** could not be established, as no relevant long range ^1^H-^13^C correlations were observed in HMBC data. Acid-mediated ester hydrolysis afforded carboxylic acids **11a** and **11b**, again characterized as an inseparable 1:0.3 mixture. HMBC data obtained for this isomeric mixture however was able to establish that the major regio-isomer was **11a** as shown. Thus correlations observed between the major isomer H-8 resonance (δ_H_ 7.84) to quinonoid resonance δ_C_ 171.7 (C-9) and from the thiazine NH (δ_H_ 9.31) to a second quinonoid resonance δ_C_ 173.1 (C-5) confirmed the identity of **11a**. In the case of base hydrolysis/autoxidation, reaction of the isomeric mixture **10a**/**10b** with 1N NaOH in a biphasic reaction in EtOAc, yielded the expected Δ^2(3)^ product **12**.

Column chromatography in this case was successful in affording the major regio-isomeric product in pure form. HMBC data analysis, in particular the observation of correlations from H-2 (δ_H_ 6.57) and H-8 (δ_H_ 7.82) to the same quinonoid carbon resonance at δ_C_ 175.2 (C-9) established the dioxothiazine ring regiochemistry of **12** as shown.

**Scheme 3:**
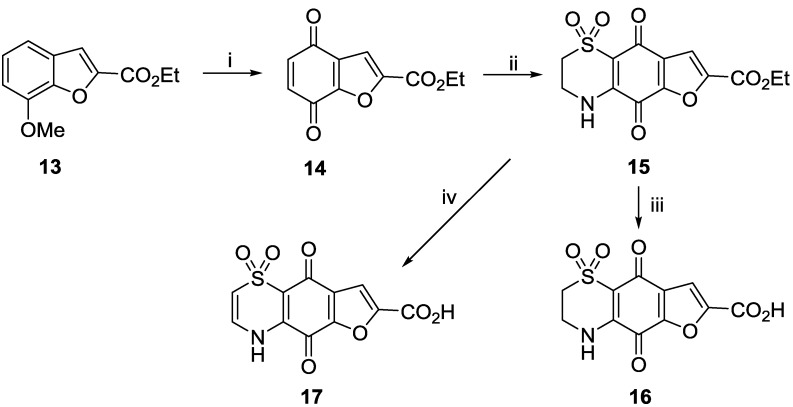
Preparation of furan analogues **15**–**17**. *Reagents and conditions*: (i) (NH_4_)_4_Ce(SO_4_)_4_·2H_2_O, MeCN/H_2_SO4, 60 °C, 90 min, 85% yield; (ii) Hypotaurine (1 equiv.), CeCl_3_·7H_2_O (0.5 equiv.), MeCN/EtOH, rt, 1 days, 43% yield; (iii) conc. HCl, 100 °C, 2 h, 63% yield; (iv) 1 N NaOH, EtOAc, rt, 2 h, 47% yield.

A series of furan analogues were prepared in analogous fashion, this time starting from commercially available 7-methoxy-benzofuran-2-carboxylic acid ethyl ester **13** (Scheme 3). Oxidation using acidified ceric ammonium sulfate afforded quinone **14** in 85% yield. Slow addition of hypotaurine to the quinone afforded a single regio-isomer **15** of the expected dioxothiazine product (43% yield). As demonstrated earlier, acidic hydrolysis of ester **15** yielded the carboxylic acid **16** (63% yield), while biphasic 1N NaOH/EtOAc hydrolysis and autoxidation afforded the Δ^2(3)^ carboxylic acid **17** in 47% yield.

### 2.2. Biological Activities

#### 2.2.1. *In Vitro* Biological Evaluation

The library of target analogues were screened against a set of four protozoa and for cytotoxicity towards the rat skeletal myoblast cell line L6 and the results are summarized in Table 1 (amide and oxidized analogues of **2**) and Table 2 (thiophene and ester analogues). All of the amide analogues **7a**–**h**,**j** evaluated were either equipotent or slightly more active against *P. falciparum* than the natural product **2**. A similar observation was made for activities towards *T. brucei rhodesiense*, except for propargyl **7h** (Table 1, entry 11) and glycyl ester **7j** (Table 1, entry 12) both of which were significantly less active than **2**. Notable in this series, unfortunately, was the lack of selectivity with most analogues exhibiting selectivity indices (SI) of 40 or less. Of this sub-set, only phenethyl amide **7e** (Table 1, entry 8) exhibited anti-protozoal activity and cytotoxic selectivity similar to those observed for **2**. The corresponding Δ^2(3)^ analogues **8a**–**k** while being typically equipotent or slightly more active against *P. falciparum*, were on the whole more cytotoxic with low selectivity. Significant amongst the series were the Δ^2(3)^ phenethyl amide **8e** (Table 1, entry 17), which was the most active anti-*T. brucei rhodesiense* analogue, and ether **8i**, ester **8j** and carboxylic acid **8k** (Table 1, entries 21–23) which maintained the anti-*Pf* activity of **2** but with modestly enhanced selectivity. There was no apparent correlation between calculated logP and observed biological activity (Table 1).

**Table 2:** Anti-protozoal and cytotoxic activities of **10a**/**10b**, **11a**/**11b**, **12**, **15**–**17**.

Entry	Compound	IC_50_ (μM) ^a^					SI *Pf* ^g^	clogP ^h^
		*T. b. rhod.* ^b^	*T. cruzi* ^c^	*L. don.* ^d^	*P. falc.* K1 ^e^	L6 ^f^		
1	**10a/10b**	0.39	0.51	6.3	0.028	0.52	18	0.1 ± 0.8
2	**11a/11b**	5.4	>290	260	0.086	230	2700	−0.2 ± 0.7
3	**12**	2.2	210	>290	0.035	140	4000	−0.2 ± 0.7
4	**15**	1.1	4.7	40	0.11	5.1	46	−0.1 ± 0.8
5	**16**	7.5	>300	>300	0.12	290	2400	−0.8 ± 0.7
6	**17**	2.7	>300	120	0.34	190	560	−0.8 ± 0.9
	Melarsoprol ^i^	0.01						
	Benznidazole ^i^		1.4					
	Miltefosine ^i^			0.53				
	Chloroquine ^i^				0.28			
	Podophyllotoxin ^i^					0.019		

^a^ IC_50_ values reported are the average of two independent assays. Assay protocols are described in [5]; ^b^ *Trypanosoma brucei rhodesiense*, STIB 900 strain, trypomastigotes stage; ^c^ *Trypanosoma cruzi*, Tulahuen C4 strain, amastigotes stage; ^d^ *Leishmania donovani*, MHOM-ET-67/L82 strain, amastigote/axenic stage; ^e^ *Plasmodium falciparum*, K1 strain, IEF stage; ^f^ L6 rat skeletal myoblast cell line; ^g^ Selectivity index for *P. falciparum* respectively = IC_50_ L6/IC_50_ *Pf*; ^h^ cLogP calculated using ALOGPS 2.1, as described in [17,18]; ^i^ Melarsoprol, benznidazole, miltefosine, chloroquine and podophyllotoxin were used as positive controls.

Thiophene and furan analogues **10a**/**10b**, **11a**/**11b**, **12**, and **15**–**17** were evaluated against the same selection of protozoa and for cytotoxicity (Table 2). Potent anti-*Pf* activity was observed for the thiophene examples, with the isomerically pure carboxylic acid **12** (Table 2, entry 3) showing a desirable combination of nanomolar potency (*Pf* IC_50_ 35 nM) and excellent selectivity (SI *Pf* 4000). The furan analogues **15**–**17** (Table 2, entries 4–6) were slightly less active towards *P. falciparum*, exhibiting IC_50_′s in the 110–340 nm range, with carboxylic acid **16** (Table 2, entry 5) exhibiting the best selectivity (SI *Pf* 2400). It is interesting to note the broad-range activities of esters **10a**/**10b** (Table 2, entry 1) and **15** (Table 2, entry 4): such pan-panel activity suggests the presence of an underlying general cytotoxic mechanism for these analogues. Once again, there was no apparent correlation between biological activity and calculated logP values.

#### 2.2.2. *In Vivo* Anti-Malarial Evaluation

Furan carboxylic acid analogue **16** was selected for preliminary proof-of-principle *in vivo* evaluation in *Plasmodium berghei* infected mice. Preliminary ip acute toxicity of **16** showed no toxicity up to the highest test dose of 150 mg/kg. Using a standard test protocol [23], a repeated ip dose of 50 (mg/kg)/day for four days led to an 85.7% reduction in parasitaemia, and an increase in mean survival time from 4–6 days (untreated control) to 9.6 days. Switching to an oral dosing experiment (100 mg/kg once per day for 4 days) yielded a 47% reduction of parasitaemia. Although not considered significant, these levels of activity for both ip and po dosing clearly identifies heterocyclic dioxothiazinoquinone carboxylic acids to be a novel anti-malarial drug scaffold warranting further structure-activity relationship studies.

## 3. Experimental Section

### 3.1. General

HRMS data were acquired on a Bruker micrOTOF-QII mass spectrometer. Infrared spectra were recorded on a Perkin-Elmer Spectrum 100 Fourier-transform IR spectrometer equipped with a universal ATR accessory. Melting points were obtained on an Electrothermal melting point apparatus and are uncorrected. NMR spectra were recorded using either a Bruker Avance DRX 300 or 400 spectrometer operating at 300 MHz or 400 MHz for ^1^H nuclei and 75 MHz or 100 MHz for ^13^C nuclei. Resonance assignments were made by interpretation of 2D data. NMR assignments marked by an asterisk are interchangeable. Proto-deutero solvent signals were used as internal references (DMSO-*d*_6_: δ_H_ 2.50, δ_C_ 39.52; CDCl_3_: δ_H_ 7.25, δ_C_ 77.0; CD_3_OD: δ_H_ 3.30, δ_C_ 49.05). Flash column chromatography was performed using reversed-phase Merck Lichroprep RP-18, or Kieselgel 60 PF silica gel. Thin layer chromatography used 0.2 mm thick plates of Kiesegel F_254_ (Merck, Manakau, New Zealand). The syntheses of **2**–**4** [16] and **9** [22] have been reported previously.

### 3.2. Synthetic Procedures

#### 3.2.1. General Procedure for the Preparation of 8-Hydroxyquinoline-2-carboxamides **5a**–**5j**

To a solution of 8-hydroxyquinoline-2-carboxylic acid and PyBOP (1.25 equiv.) in dry DMF (3–6 mL), amine (1–2 equiv.) and triethylamine (1.25 equiv.) were added under N_2_. The reaction mixture was then stirred under N_2_ at rt for 12 h, after which time the mixture was dried in vacuo. The residue was purified by reversed-phase C_18_ flash column chromatography (0%–80% MeOH in H_2_O (0.05% TFA)) and silica gel column chromatography (0%–1% MeOH in CH_2_Cl_2_).

##### 3.2.1.1. *N*-*n*-Butyl-8-hydroxyquinoline-2-carboxamide (**5a**)

From 8-hydroxyquinoline-2-carboxylic acid (100 mg, 0.529 mmol), PyBOP (330 mg, 0.64 mmol), *n*-butylamine (104 µL, 1.05 mmol) and triethylamine (88 µL, 0.632 mmol) in DMF (6 mL) to give **5a** as a yellow oil (108 mg, 84% yield).

*R_f_* = 0.68 (1% MeOH/CH_2_Cl_2_); IR ν_max_ (ATR) 3291, 1650, 1539, 1502, 1465, 1159 cm^−1^; ^1^H NMR (CDCl_3_, 400 MHz) δ_H_ 8.34 (1H, d, *J* = 8.4 Hz, H-3), 8.29 (1H, d, *J* = 8.4 Hz, H-4), 8.01 (1H, br s, NH-2′), 7.86 (1H, br s, OH), 7.53 (1H, t, *J* = 7.8 Hz, H-6), 7.40 (1H, d, *J* = 8.4 Hz, H-5), 7.23 (1H, d, *J* = 8.0 Hz, H-7), 3.53 (2H, dt, *J* = 7.2, 7.2 Hz, H_2_-3′), 1.63 (2H, p, *J* = 7.3 Hz, H_2_-4′), 1.40 (2H, sex., *J* = 7.6 Hz, H_2_-5′), 0.92 (3H, t, *J* = 7.6 Hz, H_3_-6′); ^13^C NMR (CDCl_3_, 100 MHz) δ_C_ 164.1 (C-1′), 152.2 (C-8), 148.2 (C-2), 137.8 (C-4), 136.1 (C-8a), 129.7 (C-4a), 129.2 (C-6), 119.9 (C-3), 118.3 (C-5), 111.2 (C-7), 39.5 (C-3′), 31.8 (C-4′), 20.2 (C-5′), 13.8 (C-6′); (+)-ESIMS *m/z* 245 [M + H]^+^; (+)-HRESIMS *m/z* 245.1287 [M + H]^+^ (calcd. for C_14_H_17_N_2_O_2_, 245.1285).

##### 3.2.1.2. *N*-*n*-Pentyl-8-hydroxyquinoline-2-carboxamide (**5b**)

From 8-hydroxyquinoline-2-carboxylic acid (50 mg, 0.26 mmol), PyBOP (165 mg, 0.32 mmol), *n*-pentylamine (61 µL, 0.53 mmol) and triethylamine (44 µL, 0.32 mmol) in DMF (3 mL) to give **5b** as a colorless oil (65 mg, 97% yield).

*R_f_* = 0.65 (5% MeOH/CH_2_Cl_2_); IR ν_max_ (ATR) 3266, 2929, 1647, 1500 cm^−1^; ^1^H NMR (CDCl_3_, 400 MHz) δ_H_ 8.46 (1H, t, *J* = 5.8 Hz, NH-2′), 8.33 (1H, d, *J* = 8.6 Hz, H-3), 8.24 (1H, d, *J* = 8.6 Hz, H-4), 7.49 (1H, t, *J* = 8.2 Hz, H-6), 7.34 (1H, d, *J* = 8.2 Hz, H-5), 7.20 (1H, d, *J* = 8.2 Hz, H-7), 3.46 (2H, dt, *J* = 7.4, 5.8 Hz, H_2_-3′), 1.58 (2H, p, *J* = 7.4 Hz, H_2_-4′), 1.30–1.18 (4H, m, H_2_-5′/H_2_-6′), 0.81 (3H, t, *J* = 7.4 Hz, H_3_-7′); ^13^C NMR (CDCl_3_, 100 MHz) δ_C_ 164.4 (C-1′), 152.4 (C-8), 148.0 (C-2), 137.6 (C-4), 136.6 (C-8a), 129.7 (C-4a), 129.2 (C-6), 119.7 (C-3), 118.1 (C-5), 111.2 (C-7), 39.8 (C-3′), 29.4 (C-4′), 29.1 (C-5′), 22.3 (C-6′), 13.8 (C-7′); (+)-ESIMS *m/z* 281 [M + Na]^+^; (+)-HRESIMS *m/z* [M + Na]^+^ 281.1259 (calcd. for C_15_H_18_N_2_NaO_2_, 281.1260).

##### 3.2.1.3. *N*-*n*-Octyl-8-hydroxyquinoline-2-carboxamide (**5c**)

From 8-hydroxyquinoline-2-carboxylic acid (50 mg, 0.26 mmol), PyBOP (165 mg, 0.32 mmol), *n*-octylamine (87 µL, 0.527 mmol) and triethylamine (44 µL, 0.32 mmol) in DMF (3 mL) to give **5c** as a colorless oil (73 mg, 94% yield).

*R_f_* = 0.80 (5% MeOH/CH_2_Cl_2_); IR ν_max_ (ATR) 3297, 2924, 1648, 1501 cm^−1^; ^1^H NMR (CDCl_3_, 400 MHz) δ_H_ 8.69 (1H, t, *J* = 5.7 Hz, NH-2′), 8.33 (1H, d, *J* = 8.6 Hz, H-3), 8.22 (1H, d, *J* = 8.6 Hz, H-4), 7.48 (1H, t, *J* = 8.0 Hz, H-6), 7.32 (1H, d, *J* = 8.0 Hz, H-5), 7.18 (1H, d, *J* = 8.0 Hz, H-7), 3.45 (2H, dt, *J* = 7.2, 5.7 Hz, H_2_-3′), 1.55 (2H, p, *J* = 7.2 Hz, H_2_-4′), 1.27–1.09 (10H, m, H_2_-5′/H_2_-6′/H_2_-7′/H_2_-8′/H_2_-9′), 0.80 (3H, t, *J* = 7.2 Hz, H_3_-10′); ^13^C NMR (CDCl_3_, 100 MHz) δ_C_ 164.6 (C-1′), 152.5 (C-8), 147.8 (C-2), 137.6 (C-4), 136.6 (C-8a), 129.6 (C-4a), 129.2 (C-6), 119.6 (C-3), 118.1 (C-5), 111.2 (C-7), 39.9 (C-3′), 31.7 (C-6′*), 29.6 (C-4′), 29.2 (C-7′*), 29.1 (C-8′*), 27.0 (C-5′), 22.5 (C-9′*), 14.0 (C-10′); (+)-ESIMS *m/z* 323 [M + Na]^+^; (+)-HRESIMS *m/z* [M + Na]^+^ 323.1740 (calcd. for C_18_H_24_N_2_NaO_2_, 323.1730).

##### 3.2.1.4. *N*-Benzyl-8-hydroxyquinoline-2-carboxamide (**5d**)

From 8-hydroxyquinoline-2-carboxylic acid (50 mg, 0.26 mmol), PyBOP (165 mg, 0.32 mmol), benzylamine (58 µL, 0.53 mmol) and triethylamine (44 µL, 0.32 mmol) in DMF (3 mL) to give **5d** as a colorless oil (57 mg, 79% yield).

*R_f_* = 0.72 (5% MeOH/CH_2_Cl_2_); IR ν_max_ (ATR) 3251, 3062, 1642, 1501 cm^−1^; ^1^H NMR (CDCl_3_, 400 MHz) δ_H_ 8.90 (1H, br s, NH-2′), 8.29 (1H, d, *J* = 8.4 Hz, H-3), 8.20 (1H, d, *J* = 8.4 Hz, H-4), 7.48 (1H, t, *J* = 7.9 Hz, H-6), 7.33 (1H, d, *J* = 7.9 Hz, H-5), 7.25–7.13 (6H, m, H-7/2H-5′/2H-6′/H-7′), 4.61 (2H, br s, H_2_-3′); ^13^C NMR (CDCl_3_, 100 MHz) δ_C_ 164.6 (C-1′), 152.4 (C-8), 147.5 (C-2), 137.9 (C-4′), 137.6 (C-4), 136.5 (C-8a), 129.7 (C-4a), 129.3 (C-6), 128.5 (C-5′), 127.7 (C-6′), 127.3 (C-7′), 119.7 (C-3), 118.1 (C-5), 111.3 (C-7), 43.6 (C-3′); (+)-ESIMS *m/z* 301 [M + Na]^+^; (+)-HRESIMS *m/z* [M + Na]^+^ 301.0949 (calcd. for C_17_H_14_N_2_NaO_2_, 301.0947).

##### 3.2.1.5. *N*-Phenethyl-8-hydroxyquinoline-2-carboxamide (**5e**)

From 8-hydroxyquinoline-2-carboxylic acid (50 mg, 0.26 mmol), PyBOP (165 mg, 0.32 mmol), phenethylamine (66 µL, 0.53 mmol) and triethylamine (44 µL, 0.32 mmol) in DMF (3 mL) to give **5e** as a colorless oil (65 mg, 86% yield).

*R_f_* = 0.65 (5% MeOH/CH_2_Cl_2_); IR ν_max_ (ATR) 3288, 3073, 1643, 1501 cm^−1^; ^1^H NMR (CDCl_3_, 300 MHz) δ_H_ 8.33 (1H, d, *J* = 8.5 Hz, H-3), 8.28 (1H, d, *J* = 8.5 Hz, H-4), 7.53 (1H, t, *J* = 7.8 Hz, H-6), 7.40–7.20 (7H, m, H-5/H-7/2H-6′/2H-7′/H-8′), 3.77 (2H, dt, *J* = 7.0, 6.8 Hz, H_2_-3′), 2.96 (2H, t, *J* = 7.0 Hz, H_2_-4′); ^13^C NMR (CDCl_3_, 75 MHz) δ_C_ 164.1 (C-1′), 152.3 (C-8), 147.9 (C-2), 138.8 (C-5′), 137.8 (C-4), 136.5 (C-8a), 129.7 (C-4a), 129.3 (C-6), 128.8 (C-6′), 128.7 (C-7′), 126.7 (C-8′), 119.7 (C-3), 118.1 (C-5), 111.2 (C-7), 40.7 (C-3′), 35.8 (C-4′); (+)-ESIMS *m/z* 293 [M + H]^+^; (+)-HRESIMS *m/z* [M + H]^+^ 293.1292 (calcd. for C_18_H_17_N_2_O_2_, 293.1285).

##### 3.2.1.6. *N*-(3-Phenylpropyl)-8-hydroxyquinoline-2-carboxamide (**5f**)

From 8-hydroxyquinoline-2-carboxylic acid (50 mg, 0.26 mmol), PyBOP (165 mg, 0.32 mmol), 3-phenylpropylamine (66 µL, 0.46 mmol) and triethylamine (44 µL, 0.32 mmol) in DMF (3 mL) to give **5f** as a colorless oil (67 mg, 84% yield).

*R_f_* = 0.65 (5% MeOH/CH_2_Cl_2_); IR ν_max_ (ATR) 3257, 2929, 1647, 1500 cm^−1^; ^1^H NMR (CDCl_3_, 400 MHz) δ_H_ 8.64 (1H, br s, NH-2′), 8.32 (1H, d, *J* = 8.5 Hz, H-3), 8.20 (1H, d, *J* = 8.5 Hz, H-4), 7.49 (1H, t, *J* = 7.9 Hz, H-6), 7.33 (1H, d, *J* = 7.9 Hz, H-5), 7.22–7.00 (6H, m, H-7/2H-7′/2H-8′/H-9′), 3.53–3.46 (2H, m, H_2_-3′), 2.57 (2H, t, *J* = 7.2 Hz, H_2_-5′), 1.89 (2H, p, *J* = 7.2 Hz, H_2_-4′); ^13^C NMR (CDCl_3_, 100 MHz) δ_C_ 164.6 (C-1′), 152.5 (C-8), 147.7 (C-2), 141.2 (C-6′), 137.6 (C-4), 136.5 (C-8a), 129.6 (C-4a), 129.2 (C-6), 128.2 (C-7′), 128.1 (C-8′), 125.8 (C-9′), 119.5 (C-3), 118.1 (C-5), 111.2 (C-7), 39.4 (C-3′), 33.2 (C-5′), 31.0 (C-4′); (+)-ESIMS *m/z* 329 [M + Na]^+^; (+)-HRESIMS *m/z* [M + Na]^+^ 329.1267 (calcd. for C_19_H_18_N_2_NaO_2_, 329.1260).

##### 3.2.1.7. *N*-Geranyl-8-hydroxyquinoline-2-carboxamide (**5g**)

From 8-hydroxyquinoline-2-carboxylic acid (50 mg, 0.26 mmol), PyBOP (165 mg, 0.32 mmol), geranylamine (98 µL, 0.53 mmol) and triethylamine (44 µL, 0.32 mmol) in DMF (3 mL) to give **5g** as a colorless oil (85 mg, 100% yield).

*R_f_* = 0.66 (5% MeOH/CH_2_Cl_2_); IR ν_max_ (ATR) 3276, 2914, 1646, 1501 cm^−1^; ^1^H NMR (CDCl_3_, 300 MHz) δ_H_ 8.38 (1H, t, *J* = 5.6 Hz, NH-2′), 8.33 (1H, d, *J* = 8.5 Hz, H-3), 8.23 (1H, d, *J* = 8.5 Hz, H-4), 7.49 (1H, t, *J* = 6.1 Hz, H-6), 7.34 (1H, dd, *J* = 6.1, 1.1 Hz, H-5), 7.19 (1H, dd, *J* = 6.1, 1.1 Hz, H-7), 5.27 (1H, t, *J* = 6.9 Hz, H-4′), 4.99 (1H, t, *J* = 6.9 Hz, H-8′), 4.12 (2H, dd, *J* = 6.3, 6.3 Hz, H_2_-3′), 2.04–1.95 (2H, m, H_2_-7′), 1.93–1.86 (2H, m, H_2_-6′), 1.62 (6H, s, H_3_-11′/H_3_-12′), 1.53 (3H, s, H_3_-10′); ^13^C NMR (CDCl_3_, 75 MHz) δ_C_ 164.2 (C-1′), 152.4 (C-8), 148.0 (C-2), 139.7 (C-5′), 137.6 (C-4), 136.5 (C-8a), 131.6 (C-9′), 129.6 (C-4a), 129.2 (C-6), 123.7 (C-8′), 119.7 (C-4′), 119.6 (C-3), 118.1 (C-5), 111.2 (C-7), 39.4 (C-6′), 37.7 (C-3′), 26.3 (C-7′), 25.6 (C-12′), 17.6 (C-10′), 16.3 (C-11′); (+)-ESIMS *m/z* 325 [M + H]^+^; (+)-HRESIMS *m/z* [M + H]^+^ 325.1903 (calcd. for C_20_H_25_N_2_O_2_, 325.1911).

##### 3.2.1.8. *N*-Propargyl-8-hydroxyquinoline-2-carboxamide (**5h**)

From 8-hydroxyquinoline-2-carboxylic acid (50 mg, 0.26 mmol), PyBOP (165 mg, 0.32 mmol), propargylamine (29 mg, 0.53 mmol) and triethylamine (44 µL, 0.32 mmol) in DMF (3mL) to give **5h** as a colorless oil (47 mg, 80% yield).

*R_f_* = 0.56 (10% MeOH/CH_2_Cl_2_); IR ν_max_ (ATR) 3303, 3273, 1646, 1504 cm^−1^; ^1^H NMR (DMSO-*d*_6_, 400 MHz) δ_H_ 10.17 (1H, br s, OH-9), 9.99 (1H, t, *J* = 5.8 Hz, NH-2′), 8.49 (1H, d, *J* = 8.4 Hz, H-4), 8.14 (1H, d, *J* = 8.4 Hz, H-3), 7.56 (1H, t, *J* = 7.8 Hz, H-6), 7.46 (1H, d, *J* = 7.8 Hz, H-5), 7.18 (1H, d, *J* = 7.8, Hz, H-7), 4.23 (2H, dd, *J* = 5.8, 2.4 Hz, H_2_-3′), 3.22 (1H, t, *J* = 2.4 Hz, H_2_-5′); ^13^C NMR (DMSO-*d*_6_, 100 MHz) δ_C_ 163.5 (C-1′), 153.7 (C-8), 147.0 (C-2), 137.8 (C-4), 136.4 (C-8a), 129.6 (C-4a and C-6), 118.8 (C-3), 117.5 (C-5), 111.6 (C-7), 81.1 (C-4′), 73.3 (C-5′), 28.1 (C-3′); (+)-ESIMS *m/z* 227 [M + H]^+^; (+)-HRESIMS *m/z* [M + H]^+^ 227.0814 (calcd. for C_13_H_11_N_2_O_2_, 227.0815).

##### 3.2.1.9. *N*-(2-Methoxyethyl)-8-hydroxyquinoline-2-carboxamide (**5i**)

From 8-hydroxyquinoline-2-carboxylic acid (100 mg, 0.529 mmol), PyBOP (330 mg, 0.64 mmol), 2-methoxyethylamine (92.6 µL, 1.07 mmol) and triethylamine (88 µL, 0.632 mmol) in DMF (6 mL) to give **5i** as a colorless oil (106 mg, 82% yield).

*R_f_* = 0.83 (10% MeOH/CH_2_Cl_2_); IR ν_max_ (ATR) 3315, 1648, 1542, 1501, 1120 cm^−1^; ^1^H NMR (CDCl_3_, 400 MHz) δ_H_ 8.68 (1H, br s, NH-2′), 8.38 (1H, br s, OH), 8.32 (1H, d, *J* = 8.4 Hz, H-3), 8.26 (1H, d, *J* = 8.4 Hz, H-4), 7.50 (1H, t, *J* = 8.0 Hz, H-6), 7.36 (1H, d, *J* = 8.4 Hz, H-5), 7.21 (1H, d, *J* = 7.6 Hz, H-7), 3.73 (2H, dt, *J* = 5.6, 5.2 Hz, H_2_-3′), 3.61 (2H, t, *J* = 5.2 Hz, H_2_-4′), 3.36 (3H, s, H_3_-5′); ^13^C NMR (CDCl_3_, 100 MHz) δ_C_ 164.6 (C-1′), 152.6 (C-8), 147.9 (C-2), 137.6 (C-4), 136.6 (C-8a), 129.7 (C-4a), 129.3 (C-6), 119.7 (C-3), 118.1 (C-5), 111.3 (C-7), 71.3 (C-4′), 58.8 (C-5′), 39.5 (C-3′); (+)-ESIMS *m/z* 247 [M + H]^+^; (+)-HRESIMS *m/z* 247.1076 [M + H]^+^ (calcd. for C_13_H_15_N_2_O_3_, 247.1077).

##### 3.2.1.10. *N*-Glycine(methylester)-8-hydroxyquinoline-2-carboxamide (**5j**)

From 8-hydroxyquinoline-2-carboxylic acid (100 mg, 0.529 mmol), PyBOP (330 mg, 0.64 mmol), glycine methyl ester hydrochloride (94.4 mg, 0.76 mmol) and triethylamine (220 µL, 1.58 mmol) in DMF (6 mL) to give **5j** as a colorless oil (128 mg, 93% yield).

*R_f_* = 0.86 (10% MeOH/CH_2_Cl_2_); ^1^H NMR (CDCl_3_, 400 MHz) δ_H_ 9.08 (1H, t, *J* = 6.1 Hz, NH-2′), 8.38 (1H, br s, OH), 8.03 (2H, s, H-3 and H-4), 7.43 (1H, t, *J* = 8.0 Hz, H-6), 7.23 (1H, dd, *J* = 8.4, 1.0 Hz, H-5), 7.13 (1H, dd, *J* = 7.6, 1.0 Hz, H-7), 4.27 (2H, d, *J* = 6.1 Hz, H_2_-3′), 3.72 (3H, s, H_3_-5′); ^13^C NMR (CDCl_3_, 100 MHz) δ_C_ 171.2 (C-4′), 165.0 (C-1′), 152.7 (C-8), 146.8 (C-2), 137.2 (C-4), 136.4 (C-8a), 129.6 (C-4a), 129.5 (C-6), 119.3 (C-3), 117.9 (C-5), 111.3 (C-7), 52.5 (C-5′), 41.3 (C-3′); (+)-ESIMS *m/z* 261 [M + H]^+^; (+)-HRESIMS *m/z* 261.0863 [M + H]^+^ (calcd. for C_13_H_13_N_2_O_4_, 261.0870).

#### 3.2.2. General Procedure for Preparation of Quinones **6a**–**6j**

A solution of PIFA (2–3 equiv.) in MeCN/H_2_O (2:1 mL) was cooled to 0 °C, followed by the addition of the appropriate 8-hydroxyquinoline-2-carboxamide in CH_2_Cl_2_ (1 mL). The dark brown suspension was stirred for 20 min. at 0 °C before being poured into a mixture of CH_2_Cl_2_ (20 mL) and H_2_O (30 mL). The organic phase was dried in vacuo and the crude product used in the subsequent reaction without further purification.

##### 3.2.2.1. *N*-*n*-Butyl-5,8-dioxo-5,8-dihydroquinoline-2-carboxamide (**6a**)

From *N*-*n*-butyl-8-hydroxyquinoline-2-carboxamide (**5a**) (48 mg, 0.20 mmol), PIFA (254 mg, 0.59 mmol) to give **6a** (44 mg, 85% yield) as a brown oil.

^1^H NMR (CDCl_3_, 400 MHz) δ_H_ 8.59 (1H, d, *J* = 8.0 Hz, H-3*), 8.56 (1H, d, *J* = 8.0 Hz, H-4*), 8.28 (1H, br s, NH-2′), 7.19 (1H, d, *J* = 10.4 Hz, H-7), 7.11 (1H, d, *J* = 10.4 Hz, H-6), 3.50 (2H, dt, *J* = 6.4, 6.4 Hz, H_2_-3′), 1.64 (2H, p, *J* = 7.3 Hz, H_2_-4′), 1.41 (2H, sex., *J* = 7.2 Hz, H_2_-5′), 0.94 (3H, t, *J* = 7.6 Hz, H_3_-6′); ^13^C NMR (CDCl_3_, 100 MHz) δ_C_ 183.8 (C-5), 182.6 (C-8), 162.4 (C-1′), 153.9 (C-2), 145.8 (C-8a), 139.3 (C-7*), 138.3 (C-6*), 136.5 (C-3*), 130.2 (C-4a), 126.2 (C-4*), 39.6 (C-3′), 31.6 (C-4′), 20.1 (C-5′), 13.8 (C-6′); (+)-ESIMS *m/z* 281 [M + Na]^+^; (+)-HRESIMS *m/z* 281.0893 [M + Na]^+^ (calcd. for C_14_H_14_N_2_NaO_3_, 281.0897).

##### 3.2.2.2. *N*-*n*-Pentyl-5,8-dioxo-5,8-dihydroquinoline-2-carboxamide (**6b**)

From *N*-*n*-pentyl-8-hydroxyquinoline-2-carboxamide (**5b**) (38 mg, 0.15 mmol), PIFA (127 mg, 0.30 mmol) to give **6b** (31 mg, 76% yield) as a brown oil.

^1^H NMR (CDCl_3_, 300 MHz) δ_H_ 8.57 (2H, s, H-3/H-4), 8.30 (1H, br s, NH-2′), 7.20 (1H, d, *J* = 10.3 Hz, H-7), 7.12 (1H, d, *J* = 10.3 Hz, H-6), 3.53–3.44 (2H, m, H_2_-3′), 1.71–1.61 (2H, m, H_2_-4′), 1.39–1.33 (4H, m, H_2_-5′/H_2_-6′), 0.90 (3H, t, *J* = 7.3 Hz, H_3_-7′); ^13^C NMR (CDCl_3_, 75 MHz) δ_C_ 183.7 (C-5*), 182.4 (C-8*), 162.4 (C-1′), 154.0 (C-2), 145.7 (C-8a), 139.2 (C-7), 138.2 (C-6), 136.3 (C-3*), 130.2 (C-4a), 126.1 (C-4*), 39.7 (C-3′), 29.2 (C-4′), 29.0 (C-5′), 22.2 (C-6′), 13.9 (C-7′); (+)-ESIMS *m/z* 295 [M + Na]^+^; (+)-HRESIMS *m/z* [M + Na]^+^ 295.1061 (calcd. for C_15_H_16_NaN_2_O_3_, 295.1053).

##### 3.2.2.3. *N*-*n*-Octyl-5,8-dioxo-5,8-dihydroquinoline-2-carboxamide (**6c**)

From *N*-*n*-octyl-8-hydroxyquinoline-2-carboxamide (**5c**) (73 mg, 0.24 mmol), PIFA (127 mg, 0.30 mmol) to give **6c** (64 mg, 85% yield) as a brown oil.

^1^H NMR (CDCl_3_, 300 MHz) δ_H_ 8.56 (2H, s, H-3/H-4), 8.23 (1H, br s, NH-2′), 7.18 (1H, d, *J* = 10.5 Hz, H-7), 7.10 (1H, d, *J* = 10.5 Hz, H-6), 3.48 (2H, dt, *J* = 6.7, 6.0 Hz, H_2_-3′), 1.65 (2H, p, *J* = 7.2 Hz, H_2_-4′), 1.42–1.20 (10H, br s, H_2_-5′/H_2_-6′/H_2_-7′/H_2_-8′/H_2_-9′), 0.86 (3H, t, *J* = 7.1 Hz, H_3_-10′); ^13^C NMR (CDCl_3_, 75 MHz) δ_C_ 183.8 (C-5), 182.5 (C-8), 162.4 (C-1′), 154.1 (C-2), 145.0 (C-8a), 139.3 (C-7), 138.2 (C-6), 136.4 (C-3*), 130.3 (C-4a), 126.2 (C-4*), 39.9 (C-3′), 31.8 (C-6′*), 29.6 (C-4′), 29.2 (C-7′*), 29.1 (C-8′*), 27.0 (C-5′), 22.6 (C-9′*), 14.0 (C-10′); (+)-ESIMS *m/z* 337 [M + Na]^+^; (+)-HRESIMS *m/z* [M + Na]^+^ 337.1531 (calcd. for C_18_H_22_N_2_NaO_3_, 337.1523).

##### 3.2.2.4. *N*-Benzyl-5,8-dioxo-5,8-dihydroquinoline-2-carboxamide (**6d**)

From *N*-benzyl-8-hydroxyquinoline-2-carboxamide (**5d**) (51 mg, 0.18 mmol), PIFA (127 mg, 0.30 mmol) to give **6d** (44 mg, 84% yield) as a brown oil.

^1^H NMR (CDCl_3_, 400 MHz) δ_H_ 8.67 (1H, br s, NH-2′), 8.60 (1H, d, *J* = 8.1 Hz, H-3*), 8.55 (1H, d, *J* = 8.1 Hz, H-4*), 7.36–7.26 (5H, m, 2H-5′/2H-6′/H-7′), 7.14 (1H, d, *J* = 10.6 Hz, H-7), 7.09 (1H, d, *J* = 10.6 Hz, H-6), 4.69 (2H, d, *J* = 6.3 Hz, H_2_-3′); ^13^C NMR (CDCl_3_, 100 MHz) δ_C_ 183.7 (C-5), 182.4 (C-8), 162.5 (C-1′), 153.8 (C-2), 145.8 (C-8a), 139.2 (C-7), 138.2 (C-6), 137.4 (C-4′), 136.4 (C-3*), 130.2 (C-4a), 128.7 (C-5′), 127.8 (C-6′), 127.5 (C-7′), 126.4 (C-4*), 43.6 (C-3′); (+)-ESIMS *m/z* 315 [M + Na]^+^; (+)-HRESIMS *m/z* [M + Na]^+^ 315.0748 (calcd. for C_17_H_12_N_2_NaO_3_, 315.0740).

##### 3.2.2.5. *N*-Phenethyl-5,8-dioxo-5,8-dihydroquinoline-2-carboxamide (**6e**)

From *N*-phenethyl-8-hydroxyquinoline-2-carboxamide (**5e**) (58 mg, 0.20 mmol), PIFA (127 mg, 0.30 mmol) to give **6e** (42 mg, 69% yield) as a brown oil.

^1^H NMR (CDCl_3_, 300 MHz) δ_H_ 8.57 (2H, s, H-3/H-4), 8.32 (1H, br s, NH-2′), 7.35–7.22 (5H, m, 2H-6′/2H-7′/H-8′), 7.17 (1H, d, *J* = 10.5 Hz, H-7), 7.10 (1H, d, *J* = 10.5 Hz, H-6), 3.75 (2H, dt, *J* = 7.7, 6.9 Hz, H_2_-3′), 2.98 (2H, t, *J* = 7.7 Hz, H_2_-4′); ^13^C NMR (CDCl_3_, 100 MHz) δ_C_ 183.7 (C-5), 182.3 (C-8), 162.4 (C-1′), 153.9 (C-2), 145.8 (C-8a), 139.3 (C-7), 138.2 (C-6), 137.4 (C-5′), 136.4 (C-3*), 130.2 (C-4a), 128.7 (C-6′*), 128.6 (C-7′*), 126.5 (C-8′), 126.1 (C-4*), 41.2 (C-3′), 35.8 (C-4′); (+)-ESIMS *m/z* 329 [M + Na]^+^; (+)-HRESIMS *m/z* [M + Na]^+^ 329.0904 (calcd. for C_18_H_14_N_2_NaO_3_, 329.0897).

##### 3.2.2.6. *N*-(3-Phenpropyl)-5,8-dioxo-5,8-dihydroquinoline-2-carboxamide (**6f**)

From *N*-(3-phenylpropyl)-8-hydroxyquinoline-2-carboxamide (**5f**) (65 mg, 0.21 mmol), PIFA (127 mg, 0.30 mmol) to give **6f** (45 mg, 67% yield) as a brown oil.

^1^H NMR (CDCl_3_, 400 MHz) δ_H_ 8.57 (2H, s, H-3/H-4), 8.34 (1H, br s, NH-2′), 7.33–7.19 (5H, m, 2H-7′/2H-8′/H-9′), 7.17 (1H, d, *J* = 10.4 Hz, H-7), 7.10 (1H, d, *J* = 10.4 Hz, H-6), 3.54 (2H, dt, *J* = 7.5, 6.8 Hz, H_2_-3′), 2.72 (2H, t, *J* = 7.5 Hz, H_2_-5′), 2.01 (2H, p, *J* = 7.5 Hz, H_2_-4′); ^13^C NMR (CDCl_3_, 100 MHz) δ_C_ 183.7 (C-5), 182.4 (C-8), 162.4 (C-1′), 153.9 (C-2), 145.7 (C-8a), 139.2 (C-7), 138.2 (C-6), 137.4 (C-6′), 136.4 (C-3*), 130.2 (C-4a), 128.4 (C-7′), 128.3 (C-8′), 126.1 (C-9′), 125.9 (C-4*), 39.3 (C-3′), 33.2 (C-5′), 31.0 (C-4′); (+)-ESIMS *m/z* 343 [M + Na]^+^; (+)-HRESIMS *m/z* [M + Na]^+^ 343.1063 (calcd. for C_19_H_16_N_2_NaO_3_, 343.1053).

##### 3.2.2.7. *N*-Geranyl-5,8-dioxo-5,8-dihydroquinoline-2-carboxamide (**6g**)

From *N*-geranyl-8-hydroxyquinoline-2-carboxamide (**5g**) (48 mg, 0.15 mmol), PIFA (127 mg, 0.30 mmol) to give **6g** (35 mg, 69% yield) as a brown oil.

^1^H NMR (CDCl_3_, 400 MHz) δ_H_ 8.57 (1H, d, *J* = 8.1 Hz, H-3), 8.54 (1H, d, *J* = 8.1 Hz, H-4), 8.18 (1H, br s, NH-2′), 7.19 (1H, d, *J* = 10.5 Hz, H-7), 7.09 (1H, d, *J* = 10.5 Hz, H-6), 5.28 (1H, t, *J* = 5.6 Hz, H-4′), 5.06 (1H, t, *J* = 6.8 Hz, H-8′), 4.10 (2H, dd, *J* = 6.4, 5.6 Hz, H_2_-3′), 2.11–2.05 (2H, m, H_2_-7′), 2.04–1.99 (2H, m, H_2_-6′), 1.72 (3H, s, H_3_-11′), 1.65 (3H, s, H_3_-12′), 1.58 (3H, s, H_3_-10′); ^13^C NMR (CDCl_3_, 100 MHz) δ_C_ 183.8 (C-5), 182.5 (C-8), 162.2 (C-1′), 154.1 (C-2), 145.8 (C-8a), 140.0 (C-5′), 139.3 (C-7), 138.2 (C-6), 136.3 (C-3*), 131.7 (C-9′), 130.3 (C-4a), 126.2 (C-4*), 123.8 (C-8′), 119.4 (C-4′), 39.5 (C-6′), 37.7 (C-3′), 26.3 (C-7′), 25.6 (C-12′), 17.7 (C-10′), 16.4 (C-11′); (+)-ESIMS *m/z* 361 [M + Na]^+^; (+)-HRESIMS *m/z* [M + Na]^+^ 361.1526 (calcd. for C_20_H_22_N_2_NaO_3_, 361.1523). 

##### 3.2.2.8. *N*-Propargyl-5,8-dioxo-5,8-dihydroquinoline-2-carboxamide (**6h**)

From *N*-propargyl-8-hydroxyquinoline-2-carboxamide (**5h**) (45 mg, 0.20 mmol), PIFA (229 mg, 0.53 mmol) to give **6h** (40 mg, 83% yield) as a brown oil.

^1^H NMR (CDCl_3_, 400 MHz) δ_H_ 8.57 (2H, br s, H-3/H-4), 7.19 (1H, d, *J* = 10.5 Hz, H-7), 7.12 (1H, d, *J* = 10.5 Hz, H-6), 4.30 (2H, dd, *J* = 5.6, 2.5 Hz, H_2_-3′), 2.28 (1H, t, *J* = 2.5 Hz, H-5′); ^13^C NMR (CDCl_3_, 100 MHz) δ_C_ 183.6 (C-5), 182.4 (C-8), 162.3 (C-1′), 153.3 (C-2), 145.8 (C-8a), 139.3 (C-6*), 138.3 (C-7*), 136.5 (C-3*), 130.2 (C-4a), 126.4 (C-4*), 78.9 (C-4′), 71.8 (C-5′), 29.3 (C-3′); (+)-ESIMS *m/z* 263 [M + Na]^+^; (+)-HRESIMS *m/z* [M + Na]^+^ 263.0431 (calcd. for C_13_H_8_N_2_NaO_3_, 263.0427).

##### 3.2.2.9. *N*-(2-Methoxyethyl)-5,8-dioxo-5,8-dihydroquinoline-2-carboxamide (**6i**)

From *N*-(2-methoxyethyl)-8-hydroxyquinoline-2-carboxamide (**5i**) (86 mg, 0.35 mmol), PIFA (300 mg, 0.70 mmol) to give **6i** (78 mg, 86% yield) as a brown oil.

^1^H NMR (CDCl_3_, 400 MHz) δ_H_ 8.58 (2H, s, H-3/H-4), 8.49 (1H, br s, NH-2′), 7.19 (1H, d, *J* = 10.4 Hz, H-7), 7.10 (1H, d, *J* = 10.4 Hz, H-6), 3.72 (2H, dt, *J* = 5.6, 5.2 Hz, H_2_-3′), 3.61 (2H, t, *J* = 5.2 Hz, H_2_-4′), 3.40 (3H, s, H_3_-5′); ^13^C NMR (CDCl_3_, 100 MHz) δ_C_ 183.8 (C-5), 182.3 (C-8), 162.7 (C-1′), 153.9 (C-2), 145.9 (C-8a), 139.3 (C-7*), 138.2 (C-6*), 136.4 (C-3*), 130.2 (C-4a), 126.2 (C-4*), 70.9 (C-4′), 58.9 (C-5′), 39.6 (C-3′); (+)-ESIMS *m/z* 283 [M + Na]^+^; (+)-HRESIMS *m/z* 283.0696 [M + Na]^+^ (calcd. for C_13_H_12_N_2_NaO_4_, 283.0689).

##### 3.2.2.10. *N*-Glycine(methylester)-5,8-dioxo-5,8-dihydroquinoline-2-carboxamide (**6j**)

From *N*-glycine(methylester)-8-hydroxyquinoline-2-carboxamide (**5j**) (50 mg, 0.19 mmol), PIFA (132 mg, 0.31 mmol) to give **6j** (40 mg, 77% yield) as a brown oil.

^1^H NMR (CDCl_3_, 400 MHz) δ_H_ 8.77 (2H, br t, *J* = 5.2 Hz, NH-2′), 8.59 (1H, d, *J* = 8.3 Hz, H-3), 8.55 (1H, d, *J* = 8.3 Hz, H-4), 7.20 (1H, d, *J* = 10.5 Hz, H-7), 7.13 (1H, d, *J* = 10.5 Hz, H-6), 4.31 (2H, d, *J* = 6.1 Hz, H_2_-3′), 3.79 (3H, s, H_3_-5′); ^13^C NMR (CDCl_3_, 100 MHz) δ_C_ 183.7 (C-5), 182.4 (C-8), 169.7 (C-4′), 163.1 (C-1′), 153.2 (C-2), 145.9 (C-8a), 139.4 (C-7), 138.3 (C-6), 136.5 (C-4), 130.2 (C-4a), 126.4 (C-3), 52.5 (C-5′), 41.4 (C-3′); (+)-ESIMS *m/z* 297 [M + Na]^+^; (+)-HRESIMS *m/z* 297.0477 [M + Na]^+^ (calcd. for C_13_H_10_N_2_NaO_5_, 297.0482).

#### 3.2.3. General Procedure for the Preparation of Carboxamide Analogues **7a**–**7j**

A solution of 5,8-dioxo-5,8-dihydroquinoline-2-carboxamide (**6a**–**6j**) was dissolved in MeCN/EtOH (1:1) before being cooled to 0 °C. In some cases, CeCl_3_·7H_2_O (1 equiv.) was also added to the reaction. Hypotaurine (0.8 equiv.) in H_2_O was added dropwise over 3.5 h. The reaction mixture changed color from dark brown to dark orange, and was stirred at rt for 2 days. The product was purified either by filtration and washing with H_2_O (3 × 20 mL) and MeOH (3 × 20 mL), or by reversed-phase C_18_ flash column chromatography (0%–80% MeOH in H_2_O (0.05% TFA)).

##### 3.2.3.1. *N*-*n*-Butyl-5,10-dioxo-3,4,5,10-tetrahydro-2*H*-[1,4]thiazino[2,3-*g*]quinoline-7-carboxamide 1,1-Dioxide (**7a**)

From **6a** (54 mg, 0.21 mmol) in MeCN/EtOH (1:1, 20 mL) and hypotaurine (16.0 mg, 0.15 mmol) in H_2_O (3 mL). Filtration gave **7a** as an orange powder (11.0 mg, 14% yield).

Mp 200 °C (decomp.); *R_f_* = 0.49 (10% MeOH/CH_2_Cl_2_); IR ν_max_ (ATR) 3300, 3237, 1682, 1594, 1580, 1508, 1336, 1280, 1170, 1107 cm^−1^; ^1^H NMR (DMSO-*d*_6_, 400 MHz) δ_H_ 9.35 (1H, br s, NH-4), 8.70 (1H, t, *J* = 6.4 Hz, NH-2′), 8.53 (1H, d, *J* = 8.2 Hz, H-9), 8.40 (1H, d, *J* = 8.2 Hz, H-8), 3.92–3.87 (2H, m, H_2_-3), 3.43–3.36 (obscured by solvent, H_2_-2 and H_2_-3′), 1.55 (2H, p, *J* = 7.2 Hz, H_2_-4′), 1.33 (2H, sex., *J* = 7.6 Hz, H_2_-5′), 0.91 (3H, t, *J* = 7.6 Hz, H_3_-6′); ^13^C NMR (DMSO-*d*_6_, 100 MHz) δ_C_ 176.2 (C-5), 173.4 (C-10), 162.5 (C-1′), 152.6 (C-7), 147.7 (C-4a), 145.3 (C-5a), 136.0 (C-9), 131.4 (C-9a), 126.5 (C-8), 110.7 (C-10a), 48.2 (C-2), 39.3 (obscured by solvent, C-3 and C-3′), 31.3 (C-4′), 19.6 (C-5′), 13.7 (C-6′); (+)-ESIMS *m/z* 386 [M + Na]^+^; (+)-HRESIMS *m/z* 386.0791 [M + Na]^+^ (calcd. for C_16_H_17_N_3_NaO_5_S, 386.0781).

##### 3.2.3.2. *N*-*n*-Pentyl-5,10-dioxo-3,4,5,10-tetrahydro-2*H*-[1,4]thiazino[2,3-*g*]quinoline-7-carboxamide 1,1-Dioxide (**7b**)

From **6b** (31 mg, 0.11 mmol), CeCl_3_.7H_2_O (37 mg, 98 µmol) in MeCN/EtOH (1:1, 14 mL) and hypotaurine (8.2 mg, 0.075 mmol) in H_2_O (2 mL). Filtration gave **7b** as a red-brown powder (11.0 mg, 27% yield).

Mp 200 °C (decomp.); *R_f_* = 0.44 (10% MeOH/CH_2_Cl_2_); IR ν_max_ (ATR) 3234, 2933, 1686, 1508 cm^−1^; ^1^H NMR (DMSO-*d*_6_, 400 MHz) δ_H_ 9.36 (1H, br s, NH-4), 8.72 (1H, t, *J* = 5.9 Hz, NH-2′), 8.53 (1H, d, *J* = 8.2 Hz, H-9), 8.40 (1H, d, *J* = 8.2 Hz, H-8), 3.90 (2H, br s, H_2_-3), 3.43–3.33 (4H, obscured by H_2_O, H_2_-2, H_2_-3′), 1.57 (2H, p, *J* = 6.8 Hz, H_2_-4′), 1.34–1.27 (4H, m, H_2_-5′/H_2_-6′), 0.88 (3H, t, *J* = 6.8 Hz, H_3_-7′); ^13^C NMR (DMSO-*d*_6_, 100 MHz) δ_C_ 176.2 (C-5), 173.5 (C-10), 162.5 (C-1′), 152.6 (C-7), 147.7 (C-4a), 145.4 (C-5a), 136.0 (C-9), 131.4 (C-9a), 126.5 (C-8), 110.7 (C-10a), 48.2 (C-2), 40.8 (C-3), 38.8 (C-3′), 28.8 (C-4′), 28.7 (C-5′*), 21.9 (C-6′*), 13.9 (C-7′); (+)-ESIMS *m/z* 378 [M + H]^+^; (+)-HRESIMS *m/z* [M + H]^+^ 378.1107 (calcd. for C_17_H_20_N_3_O_5_S, 378.1118).

##### 3.2.3.3. *N*-*n*-Octyl-5,10-dioxo-3,4,5,10-tetrahydro-2*H*-[1,4]thiazino[2,3-*g*]quinoline-7-carboxamide 1,1-Dioxide (**7c**)

From **6c** (32 mg, 0.10 mmol), CeCl_3_·7H_2_O (39 mg, 0.10 mmol) in MeCN/EtOH (1:1, 14 mL) and hypotaurine (9.7 mg, 0.089 mmol) in H_2_O (2 mL). Filtration and solvent wash gave **7c** as a red-brown powder (24.0 mg, 57% yield).

Mp 200 °C (decomp.); *R_f_* = 0.44 (10% MeOH/CH_2_Cl_2_); IR ν_max_ (ATR) 3240, 2925, 1669, 1521 cm^−1^; ^1^H NMR (DMSO-*d*_6_, 400 MHz) δ_H_ 9.36 (1H, br s, NH-4), 8.71 (1H, t, *J* = 6.1 Hz, NH-2′), 8.53 (1H, d, *J* = 8.0 Hz, H-9), 8.39 (1H, d, *J* = 8.0 Hz, H-8), 3.90 (2H, br s, H_2_-3), 3.41 (2H, br t, *J* = 6.1 Hz, H_2_-2), 3.32 (2H, obscured by H_2_O, H_2_-3′), 1.61–1.53 (2H, m, H_2_-4′), 1.37–1.20 (10H, m, H_2_-5′/H_2_-6′/H_2_-7′/H_2_-8′/H_2_-9′), 0.85 (3H, t, *J* = 6.7 Hz, H_3_-10′); ^13^C NMR (DMSO-*d*_6_, 100 MHz) δ_C_ 176.1 (C-5), 173.4 (C-10), 162.4 (C-1′), 152.5 (C-7), 147.6 (C-4a), 145.2 (C-5a), 135.9 (C-9), 131.3 (C-9a), 126.4 (C-8), 110.6 (C-10a), 48.1 (C-2), 39.5 (C-3/C-3′), 31.1 (C-8′), 29.0 (C-4′), 28.6 (C-6′), 26.4 (C-5′), 22.0 (C-9′), 13.9 (C-10′); (+)-ESIMS *m/z* 420 [M + H]^+^; (+)-HRESIMS *m/z* [M + H]^+^ 420.1581 (calcd. for C_20_H_26_N_3_O_5_S, 420.1588).

##### 3.2.3.4. *N*-Benzyl-5,10-dioxo-3,4,5,10-tetrahydro-2*H*-[1,4]thiazino[2,3-*g*]quinoline-7-carboxamide 1,1-Dioxide (**7d**)

From **6d** (44 mg, 0.15 mmol), CeCl_3_.7H_2_O (55 mg, 0.15 mmol) in MeCN/EtOH (1:1, 14 mL) and hypotaurine (13.0 mg, 0.12 mmol) in H_2_O (2 mL). Filtration and solvent wash gave **7d** as a red-brown powder (10.0 mg, 17% yield).

Mp 200 °C (decomp.); *R_f_* = 0.44 (10% MeOH/CH_2_Cl_2_); IR ν_max_ (ATR) 3267, 1676, 1595, 1513 cm^−1^; ^1^H NMR (DMSO-*d*_6_, 300 MHz) δ_H_ 9.37 (1H, br s, NH-4), 9.28 (1H, t, *J* = 6.2 Hz, NH-2′), 8.55 (1H, d, *J* = 8.2 Hz, H-9), 8.43 (1H, d, *J* = 8.2 Hz, H-8), 7.37–7.23 (5H, m, 2H-5′/2H-6′/H-7′), 4.57 (2H, d, *J* = 6.4 Hz, H_2_-3′), 3.90 (2H, br s, H_2_-3), 3.41 (2H, t, *J* = 6.2 Hz, H_2_-2); ^13^C NMR (DMSO-*d*_6_, 75 MHz) δ_C_ 176.2 (C-5), 173.4 (C-10), 162.8 (C-1′), 152.5 (C-7), 147.7 (C-4a), 145.5 (C-5a), 139.3 (C-4′), 136.0 (C-9), 131.5 (C-9a), 128.4 (C-5′), 127.5 (C-6′), 126.9 (C-8), 126.7 (C-7′), 110.7 (C-10a), 48.2 (C-2), 42.7 (C-3′), 39.4 (C-3); (+)-ESIMS *m/z* 420 [M + Na]^+^; (+)-HRESIMS *m/z* [M + Na]^+^ 420.0618 (calcd. for C_19_H_15_N_3_NaO_5_S, 420.0625).

##### 3.2.3.5. *N*-Phenethyl-5,10-dioxo-3,4,5,10-tetrahydro-2*H*-[1,4]thiazino[2,3-*g*]quinoline-7-carboxamide 1,1-Dioxide (**7e**)

From **6e** (22.8 mg, 0.075 mmol), CeCl_3_.7H_2_O (51 mg, 0.14 mmol) in MeCN/EtOH (1:1, 14 mL) and hypotaurine (12.0 mg, 0.11 mmol) in H_2_O (2 mL). Filtration and solvent wash gave **7e** as a red-brown powder (15.0 mg, 49% yield).

Mp 240 °C (decomp.); *R_f_* = 0.48 (10% MeOH/CH_2_Cl_2_); IR ν_max_ (ATR) 3230, 1678, 1580, 1555 cm^−1^; ^1^H NMR (DMSO-*d*_6_, 300 MHz) δ_H_ 9.37 (1H, br s, NH-4), 8.77 (1H, t, *J* = 5.9 Hz, NH-2′), 8.54 (1H, d, *J* = 8.1 Hz, H-9), 8.40 (1H, d, *J* = 8.1 Hz, H-8), 7.33–7.19 (5H, m, 2H-6′/2H-7′/H-8′), 3.93–3.87 (2H, m, H_2_-3), 3.60 (2H, dt, *J* = 7.7, 5.9 Hz, H_2_-3′), 3.40 (2H, br t, *J* = 5.4 Hz, H_2_-2), 2.90 (2H, t, *J* = 7.7 Hz, H_2_-4′); ^13^C NMR (DMSO-*d*_6_, 75 MHz) δ_C_ 176.2 (C-5), 173.4 (C-10), 162.5 (C-1′), 152.4 (C-7), 147.7 (C-4a), 145.3 (C-5a), 139.2 (C-5′), 136.0 (C-9), 131.4 (C-9a), 128.6 (C-6′), 128.4 (C-7′), 126.5 (C-8′), 126.2 (C-8), 110.7 (C-10a), 48.2 (C-2), 40.7 (C-3′), 38.6 (C-3), 35.1 (C-4′); (+)-ESIMS *m/z* 434 [M + Na]^+^; (+)-HRESIMS *m/z* [M + Na]^+^ 434.0768 (calcd. for C_20_H_17_N_3_NaO_5_S, 434.0781).

##### 3.2.3.6. *N*-(3-Phenylpropyl)-5,10-dioxo-3,4,5,10-tetrahydro-2*H*-[1,4]thiazino[2,3-*g*] quinoline-7-carboxamide 1,1-Dioxide (**7f**)

From **6f** (39.0 mg, 0.12 mmol), CeCl_3_.7H_2_O (51.0 mg, 0.14 mmol) in MeCN and EtOH (1:1, 14 mL) and hypotaurine (12.0 mg, 0.11 mmol) in H_2_O (2 mL). Filtration and solvent wash gave **7f** as a red-brown powder (29.0 mg, 57% yield).

Mp 204 °C (decomp.); *R_f_* = 0.52 (10% MeOH/CH_2_Cl_2_); IR ν_max_ (ATR) 3247, 2922, 1670, 1528 cm^−1^; ^1^H NMR (DMSO-*d*_6_, 300 MHz) δ_H_ 9.36 (1H, br s, NH-4), 8.77 (1H, t, *J* = 6.0 Hz, NH-2′), 8.54 (1H, d, *J* = 8.1 Hz, H-9), 8.40 (1H, d, *J* = 8.1 Hz, H-8), 7.31–7.15 (5H, m, H-7′/H-8′/H-9′), 3.90 (2H, t, *J* = 5.5 Hz, H_2_-3), 3.44–3.35 (4H, m, H_2_-2/H_2_-3′), 2.64 (2H, t, *J* = 7.8 Hz, H_2_-5′), 1.89 (2H, p, *J* = 7.8 Hz, H_2_-4′); ^13^C NMR (DMSO-*d*_6_, 75 MHz) δ_C_ 176.2 (C-5), 173.5 (C-10), 162.6 (C-1′), 152.6 (C-7), 147.7 (C-4a), 145.4 (C-5a), 141.6 (C-6′), 136.0 (C-9), 131.4 (C-9a), 128.3 (C-7′/C-8′), 126.6 (C-9′), 125.8 (C-8), 110.7 (C-10a), 48.2 (C-2), 39.1 (C-3), 38.8 (C-3′), 32.6 (C-5′), 30.8 (C-4′); (+)-ESIMS *m/z* 426 [M + H]^+^; (+)-HRESIMS *m/z* [M + H]^+^ 426.1119 (calcd. for C_21_H_20_N_3_O_5_S, 426.1118).

##### 3.2.3.7. (E)-*N*-(3,7-Dimethylocta-2,6-dien-1-yl)-5,10-dioxo-3,4,5,10-tetrahydro-2*H*-[1,4]thiazino [2,3-*g*]quinoline-7-carboxamide 1,1-Dioxide (**7g**)

From **6g** (26.6 mg, 0.079 mmol), CeCl_3_.7H_2_O (31 mg, 0.083 mmol) in MeCN/EtOH (1:1, 14 mL) and hypotaurine (7.2 mg, 0.066 mmol) in H_2_O (2 mL). Filtration and solvent wash gave **7g** as a dark orange powder (10.0 mg, 29% yield).

Mp 200 °C (decomp.); *R_f_* = 0.45 (10% MeOH/CH_2_Cl_2_); IR ν_max_ (ATR) 3230, 3076, 1693, 1561 cm^−1^; ^1^H NMR (DMSO-*d*_6_, 300 MHz) δ_H_ 9.35 (1H, br s, NH-4), 8.73 (1H, t, *J* = 5.7 Hz, NH-2′), 8.53 (1H, d, *J* = 8.1 Hz, H-9), 8.41 (1H, d, *J* = 8.1 Hz, H-8), 5.27 (1H, t, *J* = 6.2 Hz, H-4′), 5.07 (1H, t, *J* = 6.2 Hz, H-8′), 3.97 (2H, dd, *J* = 6.2, 5.7 Hz, H_2_-3′), 3.90 (2H, br t, *J* = 5.5 Hz, H_2_-3), 3.41 (2H, t, *J* = 5.5 Hz, H_2_-2), 2.09–2.02 (2H, m, H_2_-7′), 2.01–1.95 (2H, m, H_2_-6′), 1.71 (3H, s, H_3_-11′), 1.62 (3H, s, H_3_-12′), 1.56 (3H, s, H_3_-10′); ^13^C NMR (DMSO-*d*_6_, 75 MHz) δ_C_ 176.2 (C-5), 173.4 (C-10), 162.3 (C-1′), 152.6 (C-7), 147.7 (C-4a), 145.3 (C-5a), 137.5 (C-5′), 136.0 (C-9), 131.4 (C-9a), 130.9 (C-9′), 126.5 (C-8), 123.9 (C-8′), 121.0 (C-4′), 110.7 (C-10a), 48.2 (C-2), 40.3 (C-3), 38.6 (C-6′), 37.1 (C-3′), 25.9 (C-7′), 25.5 (C-12′), 17.5 (C-10′), 16.1 (C-11′); (+)-ESIMS *m/z* 466 [M + Na]^+^; (+)-HRESIMS *m/z* [M + Na]^+^ 466.1395 (calcd. for C_22_H_25_N_3_NaO_5_S, 466.1407).

##### 3.2.3.8. *N*-(Prop-2-yn-1-yl)-5,10-dioxo-3,4,5,10-tetrahydro-2*H*-[1,4]thiazino[2,3-*g*]quinoline-7-carboxamide 1,1-Dioxide (**7h**)

From **6h** (35 mg, 0.15 mmol), CeCl_3_.7H_2_O (46.5 mg, 0.12 mmol) in MeCN/EtOH (1:1, 14 mL) and hypotaurine (9.5 mg, 0.087 mmol) in H_2_O (2 mL). The crude reaction mixture was purified by reversed-phase C_18_ flash column chromatography to give **7h** as a bright yellow powder (15.0 mg, 29% yield).

Mp 280 °C (decomp.); *R_f_* = 0.52 (10% MeOH/CH_2_Cl_2_); IR ν_max_ (ATR) 3369, 3255, 2936, 1667, 1595 cm^−1^; ^1^H NMR (DMSO-*d*_6_, 300 MHz) δ_H_ 9.38 (1H, t, *J* = 5.4 Hz, NH-4), 9.09 (1H, t, *J* = 6.0 Hz, NH-2′), 8.55 (1H, d, *J* = 8.1 Hz, H-9), 8.41 (1H, d, *J* = 8.1 Hz, H-8), 4.13 (2H, dd, *J* = 6.0, 2.4 Hz, H_2_-3′), 3.93–3.87 (2H, m, H_2_-3), 3.40 (2H, obscured by water, H_2_-2), 3.12 (1H, t, *J* = 2.4 Hz, H-5′); ^13^C NMR (DMSO-*d*_6_, 75 MHz) δ_C_ 176.1 (C-5), 173.3 (C-10), 162.6 (C-1′), 152.0 (C-7), 147.7 (C-4a), 145.5 (C-5a), 136.0 (C-9), 131.5 (C-9a), 126.7 (C-8), 110.7 (C-10a), 80.9 (C-4′), 72.8 (C-5′), 48.2 (C-2), 39.4 (C-3), 28.7 (C-3′); (+)-ESIMS *m/z* 368 [M + Na]^+^; (+)-HRESIMS *m/z* [M + Na]^+^ 368.0294 (calcd. for C_15_H_11_N_3_NaO_5_S, 368.0312).

##### 3.2.3.9. *N*-(2-Methoxyethyl)-5,10-dioxo-3,4,5,10-tetrahydro-2*H*-[1,4]thiazino[2,3-*g*] quinoline-7-carboxamide 1,1-Dioxide (**7i**)

From **6i** (31 mg, 0.12 mmol) in MeCN/EtOH (1:1, 20 mL) and hypotaurine (7.8 mg, 0.072 mmol) in H_2_O (3 mL). The crude reaction mixture was purified by reversed-phase C_18_ flash column chromatography to give **7i** as an orange powder (11.2 mg, 26% yield).

Mp 200 °C (decomp.); *R_f_* = 0.54 (10% MeOH/CH_2_Cl_2_); IR ν_max_ (ATR) 3546, 3251, 1673, 1594, 1581, 1556, 1339, 1122 cm^−1^; ^1^H NMR (DMSO-*d*_6_, 400 MHz) δ_H_ 9.37 (1H, br s, NH-4), 8.64 (1H, t, *J* = 5.4 Hz, NH-2′), 8.54 (1H, d, *J* = 8.0 Hz, H-9), 8.41 (1H, d, *J* = 8.0 Hz, H-8), 3.92–3.88 (2H, m, H_2_-3), 3.56–3.51 (4H, m, H_2_-3′ and H_2_-4′), 3.43–3.39 (2H, m, H_2_-2), 3.29 (3H, s, H_3_-5′); ^13^C NMR (DMSO-*d*_6_, 100 MHz) δ_C_ 176.2 (C-5), 173.4 (C-10), 162.5 (C-1′), 152.2 (C-7), 147.7 (C-4a), 145.4 (C-5a), 136.1 (C-9), 131.4 (C-9a), 126.5 (C-8), 110.7 (C-10a), 70.3 (C-4′), 57.9 (C-5′), 48.2 (C-2), 39.2 (C-3), 38.7 (C-3′); (+)-ESIMS *m/z* 388 [M + Na]^+^; (+)-HRESIMS *m/z* 388.0566 [M + Na]^+^ (calcd. for C_15_H_15_N_3_NaO_6_S, 388.0574).

##### 3.2.3.10. Methyl 2-(1,1-Dioxido-5,10-dioxo-3,4,5,10-tetrahydro-2*H*-[1,4]thiazino[2,3-*g*] quinoline-7-carboxamido)acetate (**7j**)

From **6j** (50 mg, 0.18 mmol) in MeCN/EtOH (1:1, 20 mL) and hypotaurine (11.9 mg, 0.11 mmol) in H_2_O (3 mL). Reversed-phase C_18_ flash column chromatography gave **7j** as a bright red powder (13.8 mg, 20% yield).

Mp 200 °C (decomp.); *R_f_* = 0.46 (10% MeOH/CH_2_Cl_2_); IR ν_max_ (ATR) 3576, 3335, 1748, 1666, 1594, 1581, 1557, 1346, 1271, 1212, 1164, 1115 cm^−1^; ^1^H NMR (DMSO-*d*_6_, 400 MHz) δ_H_ 9.40 (1H, br t, *J* = 3.4 Hz, NH-4), 9.08 (1H, t, *J* = 6.1 Hz, NH-2′), 8.56 (1H, d, *J* = 8.0 Hz, H-9), 8.42 (1H, d, *J* = 8.0 Hz, H-8), 4.15 (2H, d, *J* = 6.1 Hz, H-3′), 3.92–3.88 (2H, m, H_2_-3), 3.67 (3H, s, H_3_-5′), 3.43–3.40 (2H, m, H_2_-2); ^13^C (DMSO-*d*_6_, 100 MHz) δ_C_ 176.2 (C-5), 173.4 (C-10), 170.0 (C-4′), 163.0 (C-1′), 151.7 (C-7), 147.7 (C-4a), 145.6 (C-5a), 136.1 (C-9), 131.6 (C-9a), 126.6 (C-8), 110.8 (C-10a), 51.9 (C-5′), 48.2 (C-2), 41.2 (C-3′), 39.2 (C-3); (+)-ESIMS *m/z* 380 [M + H]^+^; (+)-HRESIMS *m/z* 380.0538 [M + H]^+^ (calcd. for C_15_H_14_N_3_O_7_S, 380.0547).

#### 3.2.4. General Procedure for Preparation of Δ^2(3)^ Analogues **8a**–**8i**, **8k**

Thiazine-quinoline-carboxamide (**7a**–**7j**) in DMF (1–3 mL) was stirred in 2 N NaOH (3 mL) at rt for 2 h. HCl (10% vol) was added dropwise until the reaction mixture was pH 5 and the mixture was then purified by reversed-phase C_18_ flash column chromatography (0%–10% MeOH (0.05% TFA)) to give the desired product.

##### 3.2.4.1. *N*-*n*-Butyl-5,10-dioxo-5,10-dihydro-4*H*-[1,4]thiazino[2,3-*g*]quinoline-7-carboxamide 1,1-Dioxide (**8a**)

From **7a** (34.0 mg, 0.094 mmol) using the general procedure to give **8a** as a yellow solid (13.0 mg, 38% yield).

Mp 200 °C (decomp.); *R_f_* = 0.53 (10% MeOH/CH_2_Cl_2_); IR ν_max_ (ATR) 3402, 3058, 1714, 1653, 1632, 1527, 1503, 1318, 1125 cm^−1^; ^1^H NMR (DMSO-*d*_6_, 400 MHz) δ_H_ 11.42 (1H, br s, NH-4), 8.76 (1H, t, *J* = 6.0 Hz, NH-2′), 8.58 (1H, d, *J* = 8.0 Hz, H-9), 8.43 (1H, d, *J* = 8.0 Hz, H-8), 7.17 (1H, d, *J* = 9.0 Hz, H-3), 6.62 (1H, d, *J* = 9.0 Hz, H-2), 3.38 (2H, dt, *J* = 6.9, 6.9 Hz, H_2_-3′), 1.56 (2H, p, *J* = 7.0 Hz, H_2_-4′), 1.33 (2H, sex., *J* = 7.5 Hz, H_2_-5′), 0.91 (3H, t, *J* = 7.5 Hz, H_3_-6′); ^13^C NMR (DMSO-*d*_6_, 100 MHz) δ_C_ 177.7 (C-10), 175.5 (C-5), 162.4 (C-1′), 153.3 (C-7), 145.5 (C-5a), 141.4 (C-4a), 136.1 (C-9), 130.6 (C-9a), 130.5 (C-3), 126.4 (C-8), 115.2 (C-10a), 112.0 (C-2), 38.6 (C-3′), 31.3 (C-4′), 19.6 (C-5′), 13.7 (C-6′); (+)-ESIMS *m/z* 384 [M + Na]^+^; (+)-HRESIMS *m/z* 384.0632 [M + Na]^+^ (calcd. for C_16_H_15_N_3_NaO_5_S, 384.0625).

##### 3.2.4.2. *N*-*n*-Pentyl-5,10-dioxo-5,10-dihydro-4*H*-[1,4]thiazino[2,3-*g*]quinoline-7-carboxamide 1,1-Dioxide (**8b**)

From **7b** (10.0 mg, 0.027 mmol) using the general procedure to give **8b** as a yellow solid (4.0 mg, 40% yield).

Mp 280 °C (decomp.); *R_f_* = 0.44 (10% MeOH/CH_2_Cl_2_); IR ν_max_ (ATR) 3319, 3057, 1710, 1635, 1528 cm^−1^; ^1^H NMR (DMSO-*d*_6_, 300 MHz) δ_H_ 11.44 (1H, br s, NH-4), 8.78 (1H, t, *J* = 6.1 Hz, NH-2′), 8.58 (1H, d, *J* = 8.1 Hz, H-9), 8.43 (1H, d, *J* = 8.1 Hz, H-8), 7.17 (1H, d, *J* = 8.9 Hz, H-3), 6.62 (1H, d, *J* = 8.9 Hz, H-2), 3.40–3.34 (2H, m, H_2_-3′), 1.58 (2H, p, *J* = 7.5 Hz, H_2_-4′), 1.34–1.27 (4H, m, H_2_-5′/H_2_-6′), 0.88 (3H, t, *J* = 7.5 Hz, H_3_-7′); ^13^C NMR (DMSO-*d*_6_, 75 MHz) δ_C_ 177.7 (C-10), 175.5 (C-5), 162.4 (C-1′), 153.3 (C-7), 145.6 (C-5a), 141.4 (C-4a), 136.1 (C-9), 130.6 (C-9a), 130.5 (C-3), 126.4 (C-8), 115.2 (C-10a), 112.0 (C-2), 38.8 (C-3′), 28.9 (C-4′), 28.7 (C-5′), 21.9 (C-6′), 14.0 (C-7′); (+)-ESIMS *m/z* 398 [M + Na]^+^; (+)-HRESIMS *m/z* [M + Na]^+^ 398.0776 (calcd. for C_17_H_17_N_3_NaO_5_S, 3798.0781).

##### 3.2.4.3. *N*-*n*-Octyl-5,10-dioxo-5,10-dihydro-4*H*-[1,4]thiazino[2,3-*g*]quinoline-7-carboxamide 1,1-Dioxide (**8c**)

From **7c** (12.0 mg, 0.029 mmol) using the general procedure to give **8c** as a yellow solid (6.0 mg, 50% yield).

Mp 280 °C (decomp.); *R_f_* = 0.41 (10% MeOH/CH_2_Cl_2_); IR ν_max_ (ATR) 3289, 2924, 1635, 1527 cm^−1^; ^1^H NMR (DMSO-*d*_6_, 300 MHz) δ_H_ 11.44 (1H, d, *J* = 5.3 Hz, NH-4), 8.79 (1H, t, *J* = 5.8 Hz, NH-2′), 8.58 (1H, d, *J* = 8.0 Hz, H-9), 8.43 (1H, d, *J* = 8.0 Hz, H-8), 7.17 (1H, dd, *J* = 8.8, 5.3 Hz, H-3), 6.63 (1H, d, *J* = 8.8 Hz, H-2), 3.37 (obscured by solvent, H_2_-3′), 1.60–1.52 (2H, m, H_2_-4′), 1.31–1.23 (10H, m, H_2_-5′/H_2_-6′/H_2_-7′/H_2_-8′/H_2_-9′), 0.85 (3H, t, *J* = 6.8 Hz, H_3_-10′); ^13^C NMR (DMSO-*d*_6_, 75 MHz) δ_C_ 177.7 (C-10), 175.5 (C-5), 162.4 (C-1′), 153.3 (C-7), 145.6 (C-5a), 141.4 (C-4a), 136.1 (C-9), 130.6 (C-9a), 130.5 (C-3), 126.4 (C-8), 115.2 (C-10a), 112.0 (C-2), 38.9 (C-3′), 31.3 (C-8′), 29.2 (C-4′), 28.8 (C-6′), 28.7 (C-7′), 26.5 (C-5′), 22.1 (C-9′), 14.0 (C-10′); (+)-ESIMS *m/z* 440 [M + Na]^+^; (+)-HRESIMS *m/z* [M + Na]^+^ 440.1232 (calcd. for C_20_H_23_N_3_NaO_5_S, 440.1251). 

##### 3.2.4.4. *N*-Benzyl-5,10-dioxo-5,10-dihydro-4*H*-[1,4]thiazino[2,3-*g*]quinoline-7-carboxamide 1,1-Dioxide (**8d**)

From **7d** (10.0 mg, 0.025 mmol) using the general procedure to give **8d** as a yellow solid (3.0 mg, 30% yield).

Mp 280 °C (decomp.); *R_f_* = 0.47 (10% MeOH/CH_2_Cl_2_); IR ν_max_ (ATR) 3213, 1706, 1634, 1513 cm^−1^; ^1^H NMR (DMSO-*d*_6_, 400 MHz) δ_H_ 11.41 (1H, d, *J* = 5.6 Hz, NH-4), 9.33 (1H, t, *J* = 6.3 Hz, NH-2′), 8.59 (1H, d, *J* = 8.2 Hz, H-9), 8.46 (1H, d, *J* = 8.2 Hz, H-8), 7.38–7.30 (4H, m, 2H-5′/2H-6′), 7.27–7.23 (1H, m, H-7′), 7.17 (1H, dd, *J* = 8.9, 5.6 Hz, H-3), 6.61 (1H, d, *J* = 8.9 Hz, H-2), 4.58 (2H, d, *J* = 6.3 Hz, H_2_-3′); ^13^C NMR (DMSO-*d*_6_, 100 MHz) δ_C_ 177.6 (C-10), 175.4 (C-5), 162.7 (C-1′), 153.1 (C-7), 145.6 (C-5a), 141.3 (C-4a), 139.2 (C-4′), 136.1 (C-9), 130.7 (C-9a), 130.5 (C-3), 128.3 (C-5′), 127.5 (C-6′), 126.9 (C-8), 126.6 (C-7′), 115.2 (C-10a), 112.0 (C-2), 42.7 (C-3′); (+)-ESIMS *m/z* 418 [M + Na]^+^; (+)-HRESIMS *m/z* [M + Na]^+^ 418.0470 (calcd. for C_19_H_13_N_3_NaO_5_S, 418.0468). 

##### 3.2.4.5. *N*-Phenethyl-5,10-dioxo-5,10-dihydro-4*H*-[1,4]thiazino[2,3-*g*]quinoline-7-carboxamide 1,1-Dioxide (**8e**)

From **7e** (11.0 mg, 0.027 mmol) using the general procedure to give **8e** as a yellow solid (4.0 mg, 36% yield).

Mp 290 °C (decomp.); *R_f_* = 0.47 (10% MeOH/CH_2_Cl_2_); IR ν_max_ (ATR) 3103, 3067, 1714, 1678, 1512 cm^−1^; ^1^H NMR (DMSO-*d*_6_, 300 MHz) δ_H_ 11.45 (1H, d, *J* = 5.6, NH-4), 8.85 (1H, t, *J* = 6.1 Hz, NH-2′), 8.58 (1H, d, *J* = 8.1 Hz, H-9), 8.44 (1H, d, *J* = 8.1 Hz, H-8), 7.34–7.22 (5H, m, 2H-6′/2H-7′/H-8′), 7.17 (1H, dd, *J* = 8.7, 5.6 Hz, H-3), 6.62 (1H, d, *J* = 8.7 Hz, H-2), 3.61 (2H, dt, *J* = 6.9, 6.1 Hz, H_2_-3′), 2.90 (2H, t, *J* = 6.9 Hz, H_2_-4′); ^13^C NMR (DMSO-*d*_6_, 100 MHz) δ_C_ 177.7 (C-10), 175.5 (C-5), 162.4 (C-1′), 153.0 (C-7), 145.6 (C-5a), 141.4 (C-4a), 139.3 (C-5′), 136.2 (C-9), 130.7 (C-9a), 130.5 (C-3), 128.7 (C-6′), 128.5 (C-7′), 126.4 (C-7′), 126.2 (C-8), 115.2 (C-10a), 112.0 (C-2), 40.8 (C-3′), 35.1 (C-4′); (+)-ESIMS *m/z* 432 [M + Na]^+^; (+)-HRESIMS *m/z* [M + Na]^+^ 432.0618 (calcd. for C_20_H_15_N_3_NaO_5_S, 432.0625).

##### 3.2.4.6. *N*-(3-Phenylpropyl)-5,10-dioxo-5,10-dihydro-4*H*-[1,4]thiazino[2,3-*g*]quinoline-7-carboxamide 1,1-Dioxide (**8f**)

From **7f** (20.0 mg, 0.047 mmol) using the general procedure to give **8f** as a yellow solid (6.0 mg, 30% yield).

Mp 230 °C (decomp.); *R_f_* = 0.41 (10% MeOH/CH_2_Cl_2_); IR ν_max_ (ATR) 3059, 2930, 1653, 1511 cm^−1^; ^1^H NMR (DMSO-*d*_6_, 300 MHz) δ_H_ 11.45 (1H, br s, NH-4), 8.86 (1H, t, *J* = 6.1 Hz, NH-2′), 8.58 (1H, d, *J* = 8.2 Hz, H-9), 8.44 (1H, d, *J* = 8.2 Hz, H-8), 7.31–7.15 (6H, m, H-3/2H-7′/2H-8′/H-9′), 6.63 (1H, d, *J* = 9.1 Hz, H-2), 3.40 (2H, dt, *J* = 7.5, 6.1 Hz, H_2_-3′), 2.64 (2H, t, *J* = 7.5 Hz, H_2_-5′), 1.89 (2H, p, *J* = 7.5 Hz, H_2_-4′); ^13^C NMR (DMSO-*d*_6_, 100 MHz) δ_C_ 177.7 (C-10), 175.5 (C-5), 162.5 (C-1′), 153.3 (C-7), 145.6 (C-5a), 141.6 (C-6′), 141.4 (C-4a), 136.1 (C-9), 130.6 (C-9a), 130.5 (C-3), 128.3 (C-7′/C-8′), 126.5 (C-9′), 125.8 (C-8), 115.2 (C-10a), 112.0 (C-2), 38.9 (C-3′), 32.7 (C-5′), 30.8 (C-4′); (+)-ESIMS *m/z* 446 [M + Na]^+^; (+)-HRESIMS *m/z* [M + Na]^+^ 446.0790 (calcd. for C_21_H_17_N_3_NaO_5_S, 446.0781).

##### 3.2.4.7. (*E*)-*N*-(3,7-Dimethylocta-2,6-dien-1-yl)-5,10-dioxo-5,10-dihydro-4*H*-[1,4]thiazino[2,3-g] quinoline-7-carboxamide 1,1-Dioxide (**8g**)

From **7g** (10.0 mg, 0.023 mmol) using the general procedure to give **8g** a yellow solid (5.0 mg, 50% yield).

Mp 280 °C (decomp.); *R_f_* = 0.44 (10% MeOH/CH_2_Cl_2_); ^1^H NMR (DMSO-*d*_6_, 400 MHz) δ_H_ 11.44 (1H, d, *J* = 5.7 Hz, NH-4), 8.81 (1H, t, *J* = 6.2 Hz, NH-2′), 8.58 (1H, d, *J* = 7.8 Hz, H-9), 8.44 (1H, d, *J* = 7.9 Hz, H-8), 7.17 (1H, dd, *J* = 8.6, 5.7 Hz, H-3), 6.62 (1H, d, *J* = 8.6 Hz, H-2), 5.27 (1H, t, *J* = 6.6 Hz, H-4′), 5.07 (1H, t, *J* = 7.0 Hz, H-8′), 3.98 (2H, dd, *J* = 6.2, 5.8 Hz, H_2_-3′), 2.07–2.03 (2H, m, H_2_-6′), 2.01–1.96 (2H, m, H_2_-7′), 1.71 (3H, s, H_3_-11′), 1.62 (3H, s, H_3_-12′), 1.56 (3H, s, H_3_-10′); ^13^C NMR (DMSO-*d*_6_, 100 MHz) δ_C_ 177.6 (C-10), 175.5 (C-5), 162.2 (C-1′), 153.2 (C-7), 145.6 (C-5a), 141.4 (C-4a), 137.6 (C-5′), 136.1 (C-9), 131.0 (C-9a), 130.6 (C-9′), 130.5 (C-3), 126.4 (C-8), 123.9 (C-8′), 121.1 (C-4′), 115.2 (C-10a), 112.0 (C-2), 38.9 (C-6′), 37.1 (C-3′), 26.0 (C-7′), 25.5 (C-12′), 17.6 (C-10′), 16.2 (C-11′); (+)-ESIMS *m/z* 464 [M + Na]^+^; (+)-HRESIMS *m/z* [M + Na]^+^ 464.1254 (calcd. for C_22_H_23_N_3_NaO_5_S, 464.1251).

##### 3.2.4.8. *N*-(Prop-2-yn-1-yl)-5,10-dioxo-5,10-dihydro-4*H*-[1,4]thiazino[2,3-*g*]quinoline-7-carboxamide 1,1-Dioxide (**8h**)

From **7h** (8.0 mg, 0.023 mmol) using the general procedure to give **8h** as a yellow solid (6.0 mg, 76% yield).

Mp 230 °C (decomp.); *R_f_* = 0.46 (10% MeOH/CH_2_Cl_2_); IR ν_max_ (ATR) 3310, 3058, 1636, 1509 cm^−1^; ^1^H NMR (DMSO-*d*_6_, 300 MHz) δ_H_ 11.45 (1H, d, *J* = 5.5 Hz, NH-4), 9.16 (1H, t, *J* = 6.2 Hz, NH-2′), 8.60 (1H, d, *J* = 8.2 Hz, H-9), 8.45 (1H, d, *J* = 8.2 Hz, H-8), 7.17 (1H, dd, *J* = 8.8, 5.5 Hz, H-3), 6.62 (1H, d, *J* = 8.8 Hz, H-2), 4.15 (2H, dd, *J* = 5.9, 2.5 Hz, H_2_-3′), 3.13 (1H, t, *J* = 2.5 Hz, H-5′); ^13^C NMR (CDCl_3_, 100 MHz) δ_C_ 177.6 (C-10), 175.4 (C-5), 162.5 (C-1′), 152.7 (C-7), 145.8 (C-5a), 141.4 (C-4a), 136.1 (C-9), 130.8 (C-9a), 130.5 (C-3), 126.6 (C-8), 115.3 (C-10a), 112.0 (C-2), 80.9 (C-4′), 72.9 (C-5′), 28.7 (C-3′); (+)-ESIMS *m/z* 366 [M + Na]^+^; (+)-HRESIMS *m/z* [M + Na]^+^ 366.0151 (calcd. for C_15_H_9_N_3_NaO_5_S, 366.0155).

##### 3.2.4.9. *N*-(2-Methoxyethyl)-5,10-dioxo-5,10-dihydro-4*H*-[1,4]thiazino[2,3-*g*] quinoline-7-carboxamide 1,1-Dioxide (**8i**)

From **7i** (18.8 mg, 0.052 mmol) using the general procedure to give **8i** as a yellow solid (15.6 mg, 83% yield).

Mp 200 °C (decomp.); *R_f_* = 0.54 (10% MeOH/CH_2_Cl_2_); IR ν_max_ (ATR) 3250, 3057, 1633, 1508, 1278, 1097 cm^−1^; ^1^H NMR (DMSO-*d*_6_, 400 MHz) δ_H_ 11.44 (1H, s, NH-4), 8.70 (1H, t, *J* = 5.6 Hz, H-2′), 8.59 (1H, d, *J* = 8.0 Hz, H-9), 8.45 (1H, d, *J* = 8.0 Hz, H-8), 7.17 (1H, d, *J* = 9.0 Hz, H-3), 6.62 (1H, d, *J* = 9.0 Hz, H-2), 3.59–3.50 (4H, m, H_2_-3′ and H_2_-4′), 3.29 (3H, s, H_3_-5′); ^13^C NMR (DMSO-*d*_6_, 100 MHz) δ_C_ 177.6 (C-10), 175.4 (C-5), 162.4 (C-1′), 152.8 (C-7), 145.5 (C-5a), 141.3 (C-4a), 136.2 (C-9), 130.7 (C-9a), 130.5 (C-3), 126.3 (C-8), 115.3 (C-10a), 112.0 (C-2), 70.2 (C-4′), 57.9 (C-5′), 38.4 (C-3′); (+)-ESIMS *m/z* 364 [M + H]^+^; (+)-HRESIMS *m/z* 364.0606 [M + H]^+^ (calcd. for C_15_H_14_N_3_O_6_S, 364.0598).

##### 3.2.4.10. 2-(1,1-Dioxido-5,10-dioxo-5,10-dihydro-4*H*-[1,4]thiazino[2,3-*g*]quinoline-7-carboxamido)acetic Acid (**8k**)

From **7j** (13.8 mg, 0.036 mmol) using the general procedure to give carboxylic acid **8k** as a yellow oil (8.2 mg, 62% yield).

*R_f_* = 0.20 (10% MeOH/CH_2_Cl_2_); IR *ν*_max_ (ATR) 3582, 3250, 3057, 1748, 1634, 1508, 1279, 1127 cm^−1^; ^1^H NMR (DMSO-*d*_6_, 400 MHz) δ_H_ 11.95 (1H, br s, NH-4), 9.00 (1H, t, *J* = 6.0 Hz, NH-2′), 8.60 (1H, d, *J* = 8.0 Hz, H-9), 8.45 (1H, d, *J* = 8.0 Hz, H-8), 7.18 (1H, d, *J* = 8.8 Hz, H-3), 6.62 (1H, d, *J* = 8.8 Hz, H-2), 4.07 (2H, d, *J* = 6.0 Hz, H_2_-3′); ^13^C NMR (DMSO-*d*_6_, 100 MHz) δ_C_ 177.6 (C-10), 175.5 (C-5), 170.9 (C-4′), 162.7 (C-1′), 152.4 (C-7), 145.7 (C-5a), 141.6 (C-4a), 136.2 (C-9), 130.8 (C-9a), 130.6 (C-3), 126.4 (C-8), 115.2 (C-10a), 112.0 (C-2), 41.3 (C-3′); (−)-ESIMS *m/z* 362 [M − H]^−^; (−)-HRESIMS *m/z* 362.0083 [M − H]^−^ (calcd. for C_14_H_8_N_3_O_7_S, 362.0088).

#### 3.2.5. Methyl 2-(1,1-dioxido-5,10-dioxo-5,10-dihydro-4*H*-[1,4]thiazino[2,3-*g*] quinoline-7-carboxamido)acetate (**8j**)

Thionyl chloride (8.4 µL, 0.116 mmol) was added to a solution of **8k** (7.0 mg, 0.019 mmol) in dry MeOH (3 mL) at 0 °C. The reaction mixture was stirred at that temperature for 20 min, then heated to 65 °C and stirred for an additional 2 h. The solution was then cooled to rt and loaded directly onto a C_18_ reversed-phase chromatography column. The crude material was washed with two column volumes of H_2_O and the product eluted with 100% MeOH (+0.05% TFA) to afford **8j** as a yellow oil (6.8 mg, 93% yield).

*R_f_* = 0.49 (10% MeOH/CH_2_Cl_2_); IR ν_max_ (ATR) 3247, 3056, 1753, 1635, 1508, 1273, 1128 cm^−1^; ^1^H NMR (DMSO-*d*_6_, 400 MHz) δ_H_ 11.46 (1H, br d, *J* = 5.2 Hz, NH-4), 9.14 (1H, t, *J* = 6.2 Hz, NH-2′), 8.61 (1H, d, *J* = 8.0 Hz, H-9), 8.45 (1H, d, *J* = 8.0 Hz, H-8), 7.17 (1H, dd, *J* = 8.8, 5.2 Hz, H-3), 6.63 (1H, d, *J* = 8.8 Hz, H-2), 4.16 (2H, d, *J* = 6.2 Hz, H_2_-3′), 3.68 (3H, s, H_3_-5′); ^13^C NMR (DMSO-*d*_6_, 100 MHz) δ_C_ 177.6 (C-10), 175.4 (C-5), 170.0 (C-4′), 162.9 (C-1′), 152.3 (C-7), 145.7 (C-5a), 141.4 (C-4a), 136.2 (C-9), 130.9 (C-9a), 130.4 (C-3), 126.4 (C-8), 115.3 (C-10a), 112.0 (C-2), 51.9 (C-5′), 41.3 (C-3′); (+)-ESIMS *m/z* 400 [M + Na]^+^; (+)-ESIMS *m/z* 400.0222 [M + Na]^+^ (calcd. for C_15_H_11_N_3_NaO_7_S, 400.0210).

#### 3.2.6. Methyl 5,9-Dioxo-3,4,5,9-tetrahydro-2*H*-thieno[2′,3′:4,5]benzo[1,2-*b*][1,4]thiazine-7-carboxylate 1,1-Dioxide (**10a**) and Methyl 5,9-Dioxo-2,3,5,9-tetrahydro-1*H*-thieno[3′,2′:4,5]benzo [1,2-*b*][1,4]thiazine-7-carboxylate 4,4-Dioxide (**10b**)

A solution of methyl 4,7-dioxo-4,7-dihydrobenzo[*b*]thiophene-2-carboxylate (**9**) [22] (74 mg, 0.33 mmol) and CeCl_3_·7H_2_O (124 mg, 0.33 mmol) in MeCN (10 mL) and EtOH (10 mL) was cooled to 0 °C. Hypotaurine (36 mg, 0.33 mmol) in H_2_O (2 mL) was added dropwise to the mixture leading to a color change from yellow to orange. The reaction was stirred at rt for 2 days. The residue was filtered and washed with H_2_O (3 × 20 mL) and MeOH (3 × 20 mL), to give a mixture of regioisomers (**10a**/**10b**, 1:0.3 ratio determined by NMR)) as an orange solid (20 mg, 18% yield).

Mp 280 °C (decomp.); *R_f_* = 0.36 (10% MeOH/CH_2_Cl_2_); IR ν_max_ (ATR) 3222, 3003, 1726, 1683, 1579 cm^−1^; ^1^H NMR (DMSO-*d*_6_, 400 MHz) δ_H_ 9.36 (1H, br s, NH), 8.03 (1H, s, H-8 minor isomer), 7.93 (1H, s, H-8), 3.91 (3H, s, H_3_-2′), 3.89 (3H, s, H_3_-2′ minor isomer), 3.88–3.84 (2H, m, H_2_-3), 3.37 (2H, obscured by water, H_2_-2); ^13^C NMR (DMSO-*d*_6_, 75 MHz) **10a** δ_C_ 173.1 (C-5), 171.5 (C-9), 160.8 (C-1′), 148.0 (C-4a), 142.6 (C-5a*), 141.8 (C-8a*), 141.1 (C-7), 130.2 (C-8), 109.6 (C-9a), 53.3 (C-2′), 48.1 (C-2), 39.2 (C-3); (+)-FABMS *m/z* 328 [M + H]^+^; (+)-HRFABMS *m/z* [M + H]^+^ 327.9950 (calcd. for C_12_H_10_NO_6_S_2_, 327.9950).

#### 3.2.7. 5,9-Dioxo-3,4,5,9-tetrahydro-2*H*-thieno[2′,3′:4,5]benzo[1,2-*b*][1,4]thiazine-7-carboxylic Acid 1,1-Dioxide (**11a**) and 5,9-Dioxo-2,3,5,9-tetrahydro-1*H*-thieno[3′,2′:4,5]benzo[1,2-*b*][1,4] thiazine-7-carboxylic Acid 4,4-Dioxide (**11b**)

Methyl ester (as a mixture of regioisomers) **10a**/**10b** (20.0 mg, 0.061 mmol) was dissolved in conc. HCl (3 mL), and stirred at rt for 5 h, after which time, the mixture was heated to 100 °C and stirred for a further 2 h. The crude reaction mixture was subjected to reversed-phase C_18_ column chromatography (0%–10% MeOH/H_2_O (0.05% TFA)) to give **11a**/**11b** as a mixture of regioisomers (1:0.3, 11.0 mg, 57% yield) as a bright orange solid.

Mp 200 °C (decomp.); *R_f_* = 0.25 (10% MeOH/CH_2_Cl_2_); IR ν_max_ (ATR) 3357, 3230, 1674, 1577; ^1^H NMR (DMSO-*d*_6_, 300 MHz) δ_H_ 9.42 (br s, NH minor isomer), 9.31 (1H, br s, NH), 7.93 (s, H-8 minor isomer), (7.84 (1H, s, H-8), 3.86 (2H, br s, H_2_-3), 3.40–3.34 (2H, br m, H_2_-2); ^13^C NMR (DMSO-*d*_6_, 75 MHz) **11a** δ_C_ 173.1 (C-5), 171.7 (C-9), 161.8 (C-1′), 147.9 (C-4a), 144.2 (C-7), 142.8 (C-8a), 141.2 (C-5a), 129.6 (C-8), 109.6 (C-9a), 48.1 (C-2), 39.2 (C-3); (+)-ESIMS *m/z* 336 [M + Na]^+^; (+)-HRESIMS *m/z* [M + Na]^+^ 335.9605 (calcd. for C_11_H_7_NNaO_6_S_2_, 335.9607).

#### 3.2.8. 5,9-Dioxo-5,9-dihydro-4*H*-thieno[2′,3′:4,5]benzo[1,2-*b*][1,4]thiazine-7-carboxylic Acid 1,1-Dioxide (**12**)

Thiophene methyl ester (**10a**/**10b**) (20.0 mg, 0.061 mmol) was dissolved in hot EtOAc (2 mL), followed by the addition of 1 N NaOH (1 mL). The biphasic mixture was stirred at rt for 1.5 h. HCl (10% vol) was added dropwise until the reaction mixture turned acidic. The crude mixture was subjected to reversed-phase C_18_ column chromatography (0%–10% MeOH/H_2_O (0.05% TFA)) to give **12** (single regio-isomer) (15 mg, 78% yield) as a bright orange solid.

Mp 290 °C (decomp.); *R_f_* = 0.27 (10% MeOH/CH_2_Cl_2_); IR ν_max_ (ATR) 3227, 3068, 1689, 1637, 1510; ^1^H NMR (DMSO-*d*_6_, 300 MHz) δ_H_ 11.41 (1H, br s, NH), 7.82 (1H, s, H-8), 7.13 (1H, d, *J* = 8.9 Hz, H-3), 6.57 (1H, d, *J* = 8.9 Hz, H-2); ^13^C NMR (DMSO-*d*_6_, 75 MHz) δ_C_ 175.2 (C-9), 172.5 (C-5), 161.7 (C-1′), 147.4 (C-7), 141.5 (C-5a*), 141.4 (C-4a*), 141.2 (C-8a*), 130.3 (C-3), 128.1 (C-8), 114.5 (C-9a), 112.1 (C-2); (+)-FABMS *m/z* 312 [M + H]^+^; (+)-HRFABMS *m/z* [M + H]^+^ 311.9642 (calcd. for C_11_H_6_NO_6_S_2_, 311.9637).

#### 3.2.9. Methyl 4,7-Dihydroxybenzo[*b*]thiophene-2-carboxylate (**14**)

Commercially available 7-methoxy-benzofuran-2-carboxylic acid ethyl ester (**13**) (105 mg, 0.477 mmol) in MeCN/4 N H_2_SO_4_ (20 mL/5 mL) was stirred at rt, before addition of (NH_4_)_4_Ce(SO_4_)_4_·2H_2_O (1.80 g, 3.02 mol) in 4 N H_2_SO_4_ (25 mL). The reaction mixture was heated to 60 °C for 90 min. changing the colour from orange to yellow as well as inducing the formation of a white precipitate. The reaction was cooled, filtered, and the filtrate was extracted repeatedly with CH_2_Cl_2_ (5 × 50 mL). The combined organic phases were then dried (MgSO_4_) and the solvent removed *in vacuo* to give **14** as a yellow solid (89 mg, 85% yield). The product was used immediately in the next step without further purification.

IR ν_max_ (ATR) 3570, 2955, 1752, 1726, 1534, 1475, 1367, 1187, 1160, 1139 cm^−1^; ^1^H NMR (DMSO-*d*_6_, 75 MHz) δ_H_ 7.49 (1H, s, H-3), 6.82 (2H, s, H-5/H-6), 4.45 (2H, q, *J* = 7.2 Hz, H_2_-3′), 1.42 (3H, t, *J* = 7.2 Hz, H_3_-4′); EIMS *m/z* 220 [M]^+^; (+)-HREIMS *m/z* [M]^+^ 220.0369 (calcd. for C_11_H_8_O_5_, 220.0372).

#### 3.2.10. Ethyl 5,9-Dioxo-3,4,5,9-tetrahydro-2*H*-benzofuro[5,6-*b*][1,4]thiazine-7-carboxylate 1,1-Dioxide (**15**)

A solution of quinone **14** (100 mg, 0.45 mmol) and CeCl_3_.7H_2_O (78 mg, 0.21 mmol) in MeCN (10 mL) and EtOH (10 mL) was cooled to 0 °C. Hypotaurine (49 mg, 0.45 mmol) in H_2_O (1 mL) was added dropwise to the reaction mixture, changing the colour from yellow to orange. The reaction was stirred at rt for 24 h. The residue was filtered and washed with H_2_O (3 × 20 mL) and MeOH (3 × 20 mL), to give **15** (62 mg, 43% yield) as a red solid.

Mp 277 °C; *R_f_* = 0.36 (10% MeOH/CH_2_Cl_2_); IR ν_max_ (ATR) 3225, 1733, 1695, 1566 cm^−1^; ^1^H NMR (DMSO-*d*_6_, 300 MHz) δ_H_ 9.38 (1H, br s, NH), 7.58 (1H, s, H-8), 4.37 (2H, q, *J* = 7.1 Hz, H_2_-3′), 3.85 (2H, dt, *J* = 5.7, 5.7 Hz, H_2_-3), 3.34 (2H, t, *J* = 5.7 Hz, H_2_-2), 1.33 (3H, t, *J* = 7.1 Hz, H-4′); ^13^C NMR (DMSO-*d*_6_, 75 MHz) δ_C_ 172.2 (C-9), 167.8 (C-5), 157.1 (C-1′), 149.2 (C-5a), 148.7 (C-7), 147.4 (C-4a), 130.1 (C-8a), 114.2 (C-8), 108.9 (C-9a), 62.0 (C-3′), 48.1 (C-2), 39.2 (C-3), 14.0 (C-4′); (+)-FABMS *m/z* 326 [M + H]^+^; (+)-HRFABMS *m/z* [M + H]^+^ 326.0341 (calcd. for C_13_H_12_NO_7_S, 326.0335).

#### 3.2.11. 5,9-Dioxo-3,4,5,9-tetrahydro-2*H*-benzofuro[5,6-*b*][1,4]thiazine-7-carboxylic Acid 1,1-Dioxide (**16**)

Ethyl ester **15** (64 mg, 0.20 mmol) was dissolved in conc. HCl (3 mL), and the mixture was heated to 100 °C and stirred for 2 h. The crude reaction mixture was purified by reversed-phase C_18_ column chromatography (0%–10% MeOH/H_2_O (0.05% TFA)), to give **16** (37 mg, 63% yield) as a bright red solid.

Mp 210 °C (decomp.); *R_f_* = 0.23 (10% MeOH/CH_2_Cl_2_); IR ν_max_ (ATR) 3234, 3093, 1635, 1694, 1561 cm^−1^; ^1^H NMR (DMSO-*d*_6_, 300 MHz) δ_H_ 9.35 (1H, br s, NH-4), 7.47 (1H, s, H-8), 3.87–3.82 (2H, m, H_2_-3), 3.37–3.31 (2H, m, H_2_-2); ^13^C NMR (DMSO-*d*_6_, 75 MHz) δ_C_ 172.5 (C-9), 167.8 (C-5), 158.5 (C-1′), 150.3 (C-5a*), 149.0 (C-7*), 147.4 (C-4a), 130.3 (C-8a), 113.5 (C-8), 108.9 (C-9a), 48.1 (C-2), 39.5 (C-3); (+)-ESIMS *m/z* 298 [M + H]^+^; (+)-HRESIMS *m/z* [M + H]^+^ 298.0009 (calcd. for C_11_H_8_NO_7_S, 298.0016).

#### 3.2.12. 5,9-Dioxo-5,9-dihydro-4*H*-benzofuro[5,6-*b*][1,4]thiazine-7-carboxylic Acid 1,1-Dioxide (**17**)

Ethyl ester **15** (15.0 mg, 0.046 mmol) was dissolved in hot EtOAc (2 mL), followed by the addition of 1 N NaOH (1 mL). The biphasic mixture was stirred at rt for 2 h. HCl (10% vol) was added dropwise until the reaction mixture turned acidic. The crude product was purified by reversed-phase C_18_ column chromatography (0%–10% MeOH/H_2_O (0.05% TFA)), to give **17** (6.4 mg, 47% yield) as a red solid.

Mp 280 °C (decomp.); *R_f_* = 0.36 (10% MeOH/CH_2_Cl_2_); IR ν_max_ (ATR) 3223, 3072, 1677, 1577, 1516 cm^−1^; ^1^H NMR (DMSO-*d*_6_, 400 MHz) δ_H_ 11.42 (1H, br s, NH-4), 7.47 (1H, s, H-8), 7.12 (1H, d, *J* = 8.8 Hz, H-3), 6.58 (1H, d, *J* = 8.8 Hz, H-2); ^13^C NMR (DMSO-*d_6_*, 75 MHz) δ_C_ 176.1 (C-9), 167.4 (C-5), 158.5 (C-1′), 152.0 (C-7), 149.2 (C-5a), 140.4 (C-4a), 130.2 (C-3), 129.1 (C-8a), 113.9 (C-9a), 112.3 (C-2 and C-8); (+)-FABMS *m/z* 296 [M + H]^+^; (+)-HRFABMS *m/z* [M + H]^+^ 295.9861 (calcd. for C_11_H_6_NO_7_S, 295.9865).

### 3.3. Biological Assays

#### 3.3.1. *In Vitro* Anti-Protozoal Activity

The *in vitro* activities against the protozoan parasites *T.b. rhodesiense*, *T. cruzi*, *L. donovani*, and *P. falciparum* and cytotoxicity assessment against L6 cells were determined as reported elsewhere [5]. The following strains, parasite forms and positive controls were used: *T.b. rhodesiense*, STIB900, trypomastigote forms, melarsoprol, IC_50_ of 0.01 μM (4 ng/mL); *T. cruzi*, Tulahuen C2C4, amastigote forms in L6 rat myoblasts, benznidazole, IC_50_ of 1.4 μM (0.352 μg/mL); *L. donovani*, MHOM/ET/67/L82, axenic amastigote forms, miltefosine, IC_50_ of 0.5 μM (0.213 μg/mL); *P. falciparum*, K1 (chloroquine and pyrimethamine resistant), erythrocytic stages, chloroquine, IC_50_ of 0.20 μM (0.065 μg/mL) and L6 cells, rat skeletal myoblasts, podophyllotoxin, IC_50_ of 0.01 μM (0.004 μg/mL).

#### 3.3.2. *In Vivo* Anti-Malarial Efficacy Studies

*In vivo* anti-malarial activity was assessed as previously described [23]. Groups of three female NMRI mice (20–22 g) were intravenously infected with 2 × 10^7^ parasitized erythrocytes on day 0 with GFP-transfected *P. berghei* strain ANKA [24]. Compounds were formulated in 100% DMSO, diluted 10-fold in distilled water and administered intraperitoneally in a volume of 10 ml kg^−1^ on four consecutive days (4, 24, 48 and 72 h post infection). Control experiments used DMSO-H_2_O vehicle alone. Parasitemia was determined on day 4 post infection (24 h after last treatment) by FACS analysis. Activity was calculated as the difference between the mean per cent parasitaemia for the control (*n* = 5 mice) and treated groups expressed as a per cent relative to the control group. The survival of the animals was usually monitored up to 30 days: a compound was considered curative if the animal survived to day 30 after infection with no detectable parasites. *In vivo* efficacy studies in mice were conducted according to the rules and regulations for the protection of animal rights (“Tierschutzverordnung”) of the Swiss “Bundesamt für Veterinärwesen”. They were approved by the veterinary office of Canton Basel-Stadt, Switzerland.

## 4. Conclusions

The dioxothiazinoquinone marine natural product ascidiathiazone A (**2**) has been identified as a moderate *in vitro* growth inhibitor of *Trypanosoma brucei rhodesiense* and *Plasmodium falciparum*. A series of C-7 amide and Δ^2(3)^ analogues were prepared that explored the influence of lipophilicity and oxidation state on observed anti-protozoal activity and selectivity. Little variation in anti-malarial potency was observed (IC_50_ 0.62–6.5 μM), and no correlation was apparent between anti-malarial and anti-*T. brucei* activity. Changing the quinoline-based structure of **2** to incorporate benzofuran or benzothiophene moieties yielded particularly potent anti-malarials. The finding of ip and oral dosing anti-malarial activity for benzofuran carboxylic acid **16** is highly encouraging, suggesting that future studies should be directed at exploring this novel antiprotozoal pharmacophore.
